# Systematic meta-analyses and field synopsis of genetic association studies of violence and aggression

**DOI:** 10.1038/mp.2013.31

**Published:** 2013-04-02

**Authors:** E Vassos, D A Collier, S Fazel

**Affiliations:** 1 King's College London, Institute of Psychiatry, MRC SGDP Centre, London, UK; 2 Oxford Health NHS Foundation Trust, Oxford, UK; 3 Department of Psychiatry, University of Oxford, Oxford, UK

**Keywords:** association studies, genetics, meta-analysis, aggression, violence

## Abstract

A large number of candidate gene studies for aggression and violence have been conducted. Successful identification of associations between genetic markers and aggression would contribute to understanding the neurobiology of antisocial behavior and potentially provide useful tools for risk prediction and therapeutic targets for high-risk groups of patients and offenders. We systematically reviewed the literature and assessed the evidence on genetic association studies of aggression and related outcomes in order to provide a field synopsis. We searched PubMed and Huge Navigator databases and sought additional data through reviewing reference lists and correspondence with investigators. Genetic association studies were included if outcome data on aggression or violent behavior either as a binary outcome or as a quantitative trait were provided. From 1331 potentially relevant investigations, 185 studies constituting 277 independent associations on 31 genes fulfilled the predetermined selection criteria. Data from variants investigated in three or more samples were combined in meta-analyses and potential sources of heterogeneity were investigated using subgroup analyses. In the primary analyses, which used relaxed inclusion criteria, we found no association between any polymorphism analyzed and aggression at the 5% level of significance. Subgroup analyses, including by severity of outcome, age group, characteristics of the sample and ethnicity, did not demonstrate any consistent findings. Current evidence does not support the use of such genes to predict dangerousness or as markers for therapeutic interventions.

## Introduction

Interpersonal violence is a major cause of mortality, morbidity and economic cost to society. In the Americas, it is the second highest cause of disability-adjusted living years, contributing to ∼5% of all disabilities, and sixth highest cause in young people worldwide.^1^ Its contribution to worldwide mortality and morbidity is projected to increase in the next two decades, particularly in low- and middle-income countries. In the United States, justice expenditure was $214 billion in 2006,^2^ and 3% of all medical expenses are secondary to violent crime.^3^ To date, most interventions designed to reduce violence risk have mostly small effects,^4^ suggesting that the causes of violence need further clarification if treatments are to improve.^5^

There is compelling evidence from many twin and adoption studies that a substantial proportion of human aggressive behavior is attributable to genetic variation. The heritability of antisocial personality and behavior is estimated to be ∼50–60%,^6^ human aggression ∼50%^7^ and anger control between 27 and 37%.^8^ Based on these estimates, several research groups have performed genetic association studies to identify specific genes implicated in aggressive behavior, selected on the basis of their likely involvement in relevant pathways.^9^

The selection of candidate genes has been shaped by leading theories of the neurobiology of aggression.^10, 11^ These mainly implicate the serotonergic and catecholaminergic neurobiological systems. Traditionally, the serotonergic system has been considered the primary regulator of aggressive behavior. Different lines of evidence, including reduced cerebrospinal fluid levels of the serotonin (5-HT) metabolite 5-HIAA, lower prolactin responses to challenge with 5-HT agonists in aggressive individuals and increased laboratory aggression following tryptophan depletion, have supported the link between disruption of the serotonin system and aggressiveness.^12, 13^ Genes related to the serotonergic system that have been most widely investigated are *tryptophan hydroxylase 1* (*TPH1*), *5HT receptor 1B* (*HTR1B*), *5HT receptor 2A* (*HTR2A*) and *serotonin transporter* (*5-HTT* or *SLC6A4*).

The role of catecholamines in anger and aggression has been extensively studied in human and animal experiments. Pharmacological manipulation of dopamine and noradrenalin alters aggressive behavior in animals, whereas D2 dopamine receptor antagonists have been used effectively in the treatment of aggression in certain patient groups.^10^ As the major enzymes responsible for the catabolism of catecholamines in the brain are catechol-*O*-methyltransferase (COMT) and monoamine oxidase A (MAOA), genes encoding them have been candidates for the regulation of aggressive behavior, a hypothesis that has been supported by animal studies reporting increased aggressive behavior in COMT and MAOA knockout mice.^14^ Other candidate genes that have been widely investigated include those involved in dopamine function (dopamine receptor *DRD4* and dopamine transporter gene *SLC6A3/DAT1*) and *brain-derived neurotrophic factor* (*BDNF*).^15^

As many studies have been conducted claiming or refuting the genetic association of polymorphisms in various candidate genes with aggression or violence, it is increasingly difficult for clinicians and researchers to follow and evaluate the accumulating evidence on the subject. This is also possibly becoming relevant to legal systems in some Western countries as DNA analysis has been rarely used as evidence in criminal trials,^16^ but may increase in its use in the next few years. Therefore, we systematically reviewed genetic association studies of aggression and subjected all polymorphisms studied in more than three independent samples to meta-analyses.

## Materials and methods

### Search strategy and selection criteria

**Figure 1:**
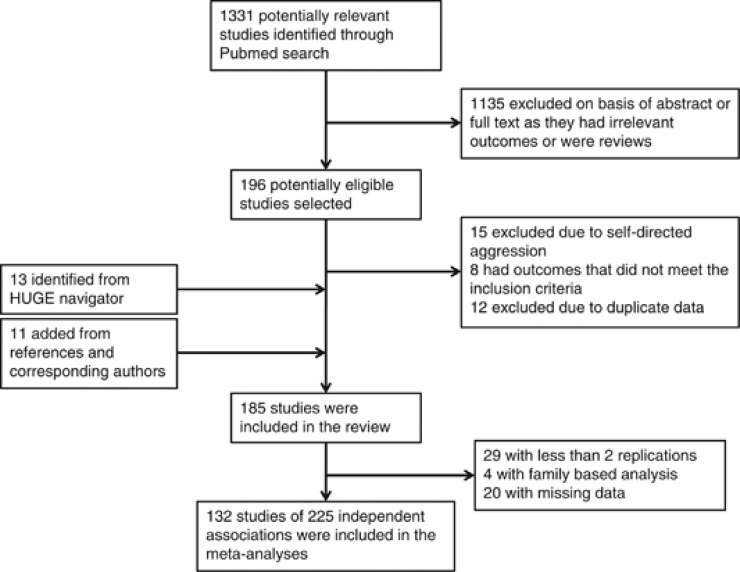
Study flow diagram.

We followed the Preferred Reporting Items for Systematic Reviews and Meta-analyses (PRISMA) Statement.^17^ PubMed was searched using the search terms ‘(aggression OR anger OR hostility OR irritability OR violence OR convict* OR crimin* OR offend*) AND (genetics OR gene OR polymorphism OR genotype OR allele OR genome OR haplotype)' in title/abstract field without language restrictions from 1 January 1966 to 31 July 2011. This search yielded 1331 papers that were screened for inclusion using the abstract or full text as necessary. In addition, we searched the Huge Navigator database (http://hugenavigator.net) for genes associated with aggression, violence or anger. We scanned reference lists of all included studies and relevant reviews for additional studies that had not been identified through the electronic search and we communicated with authors to request additional data, if available (Figure 1).^18, 19, 20, 21, 22, 23, 24, 25, 26, 27, 28, 29, 30, 31, 32, 33, 34^

Inclusion criteria for the systematic review were publications that included data on the association between genotypes or alleles and categorical measures of violence or criminality in a case–control design, or quantitative (that is, continuous) measures of aggression. The samples analyzed in the original studies were drawn from the general population or were specific subpopulations (for instance, psychiatric inpatients, alcohol or drug users, offenders, students, people with personality disorders, patients with schizophrenia, suicide attempters). No age limits were set in the primary analyses. To include the maximum data available for the initial analyses, we employed a broad definition of aggression as described below. Exclusion criteria were: self-directed aggression (for example, aggressive means of suicidal attempts) if this was the sole outcome and when trait hostility was assessed on the basis of a single question (for example, in the Positive and Negative Syndrome Scale).^35^ Studies were identified by one of the authors (EV) and then independently checked for inclusion by another (SF). Discrepancies were resolved by discussion and correspondence with authors. Detailed methods, including measures of exposure and outcome, and data management of specific genes are presented in the Supplementary Material.

### Statistical analyses

Studies with categorical and continuous outcomes of aggression were analyzed separately because the first employed case–control designs, whereas the second examined aggression in a sample as a quantitative trait. For studies with binary outcomes, we tested allelic association (log-additive genetic model), and in studies with continuous outcomes, we tested all three genetic models (dominant, recessive and additive) when sufficient data were available (Supplementary Methods). In order for a polymorphism to be included in a meta-analysis, we required ⩾3 separate samples to examine the association of this polymorphism with aggression as a quantitative trait or with violence as a binary outcome. The threshold of a minimum of three studies for the meta-analysis was selected so that there were at least two replication attempts of the original finding.^36^ However, all the primary studies that met the inclusion criteria are reported (Supplementary Table 1). Family-based association studies of related individuals were excluded. All statistical analyses were performed with STATA statistical software, release 10 (StataCorp. 2007, TX, USA) and the R Project for Statistical Computing (www.r-project.org).

Initially, we calculated summary effect size and 95% confidence intervals with the DerSimonian and Laird random effects model,^37^ a more conservative approach to pooling data that utilizes weights that incorporate both the within- and between-study variance. We estimated between-study heterogeneity by calculating Cochran's Q and the *I*^2^ statistic with its confidence intervals.^38, 39^ The latter incorporates the percentage of variation across studies due to heterogeneity.^40^ As values of *I*^2^ >50% are generally considered indicative of large heterogeneity, we repeated the analysis with fixed effect models using the Mantel–Haenszel weighting method when *I*^2^ values were <50%. However, we favored the random effects method because both Q and *I*^2^ metrics carry considerable uncertainty when few studies are included in a meta-analysis.^41, 42^

### Sensitivity analyses

As we used a broad definition of aggression and samples were heterogeneous in relation to baseline characteristics, we performed the following *post hoc* analyses: (1) excluding non-European and mixed ethnicity samples; (2) stratifying age groups (mean age <16, 16–65 and >65 years old); (3) separate analysis of samples from the general population, clinical samples with psychiatric disorder, offenders and samples selected for substance use; and (4) dividing the outcomes by severity to anger, general measures of aggressiveness including antisocial personality, history of violent acts and criminal offending.

### Assessment of epidemiological credibility

Evidence of publication bias was examined using Egger's and Begg's tests^43, 44^ in studies with continuous outcomes, and the modified version of Egger's test^45^ in studies with a binary outcome measure. For meta-analyses with nominally significant findings, the strength of cumulative evidence was graded based on the Human Genome Epidemiology Network (HuGENet) guidelines^46^ (Supplementary Table 2).

## Results

### Literature search

We identified 185 publications, reporting 277 independent association analyses in 31 different genes in over 60 000 individuals in total. These were conducted in 29 countries between 1992 and 2011. The number of participants per study varied considerably (range 21–3913, median=317). Of these studies, 92 (50%) reported a significant association of a genotype or haplotype with some aggression measure in the overall sample or a subsample. A remainder also reported some association with a secondary phenotype or significant gene–environment interaction. However, 29 studies did not have sufficient evidence of replication (according to our criteria of a minimum of two replication studies) and are not reported. Genes examined in such investigations include *HTR1A*, *HTR2C*, *ABCG1*, *ADRA2A*, *AP-2beta*, *ApoE*, *AVPR1A*, *TFAP2B*, *ESR1*, *CREB1*, *DRD1*, *DRD3*, *NOS1*, *TACR1*, *OXTR*, *TBX19*, *TH*, *CYP2D6* and *TPH-2*. A description of all eligible studies is presented in Supplementary Table 1.

### Studies with categorical outcomes

**Figure 2:**
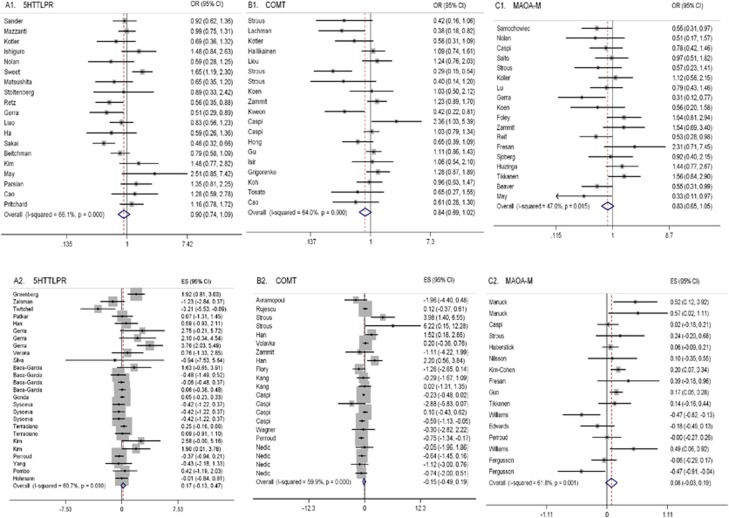
Forest plots of the association of the three most studied polymorphisms with aggression and related outcomes. The upper row (A1, B1 and C1) presents allelic associations of *5HTTLPR*, *COMT* and *MAOA* in males (*MAOA-M)* with aggression as a categorical outcome. The lower row (A2, B2 and C2) presents associations of the same polymorphisms with continuous outcomes under the additive model for *5HTTLPR* and *COMT* and with the hemizygous genotype for *MAOA* in males. 95% CI, 95% confidence interval; ES, effect size; OR, odds ratio.

Eleven polymorphisms (HTR1B-G861C, 5HTTLPR, 5HTT-VNTR, BDNF-Val66Met, COMT-Val158Met, SLC6A3 40 bpVNTR, DRD2-Taq1A, AR_(CAG)n, DRD4-ex3 48 bpVNTR, MAOA promoter 30 bpVNTR and TPH1-A779C/A218C) were meta-analyzed using random effects models. None of these variants were significantly associated with violence (Table 1 and Figure 2). The AR_(CAG)n had an elevated odds ratio of 3.04, but because of the small numbers studied and high heterogeneity, it missed the threshold for significance (*P*=0.06). Repetition of the analyses for the genetic variants with low heterogeneity using fixed effect models did not alter these overall findings. The power of the pooled samples for 5HTTLPR, COMT-Val158Met and MAOA promoters in males to detect association with odds ratio >1.1 at 0.05 level of significance was 0.87, 0.73 and 0.51, respectively. For odds ratio >1.2, the power was >95%.

### Studies with continuous outcomes

The associations of nine polymorphisms (HTR2A-1438A/G, 5HTTLPR, BDNF-Val66Met, COMT-Val158Met, SLC6A3 40 bpVNTR, DRD4-ex3 48 bpVNTR, MAOA promoter 30 bpVNTR, TPH1-A779C/A218C and AR_(CAG)n) with aggressiveness as a quantitative trait were examined with meta-analyses. For each marker, we examined the dominant, recessive and additive genetic models with the exception of DRD4-ex3 (as above), BDNF (in one of the three available studies val66 homozygous compared with met66 carriers) and the hemizygous MAOA and AR genes in males. None of the pooled estimates were significant at *P*<0.05 level (Table 2 and Figure 2). Repetition of the analyses where there was low heterogeneity with fixed effect methods did not substantially alter the results.

### Sensitivity analyses

For studies with categorical outcomes (Supplementary Figure 1), the following associations were found: (1) COMT-Val158Met was significantly associated with violence (*P*=0.02) as a categorical outcome; (2) when examining violence as a categorical outcome only, there was an association for MAOA promoter 30 bpVNTR in males with a history of violence (*P*=0.023); (3) looking at different samples separately, TPH1-A779C/A218C was associated with aggression in cases with psychiatric history (*P*=0.015). For investigations with continuous outcomes (Supplementary Figure 2), the following associations were found: (1) 5HTTLPR was associated with aggressiveness under the dominant model (long allele carriers vs short homozygous) in adults (age 16–65 years; *P*=0.024) and in substance users (*P*=0.009); (2) COMT was associated with aggressiveness under the recessive model (val homozygous vs met carriers) in Caucasian samples (*P*=0.02), in substance users (*P*=0.039) and with anger as outcome measure (*P*=0.02) and under the additive model with aggression in Caucasians (*P*=0.039) and in substance users (*P*=0.034); (3) MAOA promoter 30 bpVNTR in females under the recessive model (high activity alleles homozygous vs low activity carriers) was associated with aggressiveness as outcome measure (*P*=0.04; Table 3). The credibility of these associations as graded with the HuGENet guidelines was moderate or weak.

## Discussion

This systematic review examined genetic associations with aggression and related outcomes using data from 185 studies involving over 60 000 participants. We synthesized data on 12 polymorphisms in which there were at least two replications, and investigated sources of variation between the reported effect sizes for each polymorphism. In addition, we conducted sensitivity analyses that used more homogeneous samples and outcome measures, stratified age groups and excluded non-European samples.

Overall, we did not find any strong associations between these polymorphisms and aggression outcomes. In our primary analyses, despite the large number of comparisons, we did not find significant associations. Although there were a few significant associations at the 5% level in the sensitivity analyses, this was not unexpected because of the large number of comparisons and likely a chance finding. Even if some results reached significance with the addition of further studies, the estimated effect sizes are arguably below the levels recommended for practical significance.^47^ They would, however, be informative for understanding mechanisms and pathways.

The lack of confirmed associations with candidate genes appears to contrast with the expectations in the field, based on the confirmed heritability of aggression.^7^ Part of the explanation could be that it is unlikely that few candidate genes explain a complex behavior like aggression and many hundreds or thousands genes probably interact in a complex manner. Second, unlike clearly harmful disease phenotypes, aggression and even violence are complex behaviors, and may have adaptive function in moderate doses and challenging environments. In other words, aggression and violence exist on a continuum that makes it more likely that they are determined by many genes of moderate or small effect. Third, the sample sizes used in the reviewed studies were small and current evidence from genome-wide association studies shows that much larger samples are needed to reveal interesting findings in most human traits and diseases. This is consistent with other work that has demonstrated that pinning down specific polymorphisms possibly of small effect for complex phenotypes has been unfruitful,^48^ and that individual studies usually present false results.^49^

The lack of associations with genetic markers questions some expert opinion in the field, which has drawn on genetic evidence to support the use of selective serotonin reuptake inhibitors to treat aggression.^11, 50^ As it stands, our results suggest that the evidence of the contribution of the examined polymorphisms to aggression is at most weak and it is unlikely that they can be used to assist with violence risk prediction. Our findings also undermine the quasi-scientific basis for the increasing, although rare, use of genotypic data of criminal defendants as evidence in courts.^51, 52^

The strengths of the current review include analyzing studies with both continuous and categorical outcomes, considering aggression as a quantitative or binary trait and receiving tabular data from more than half the studies where there were missing data. The latter contributed to the large study size and the ability to study a number of separate polymorphisms with sufficient power. We examined associations with aggression and interpersonal violence, a harder outcome measure. However, as there is evidence that there are biological, sociological and psychological factors relevant to the etiology of violence,^53^ we also examined associations with aggressiveness as a personality trait, which has a potentially stronger genetic predisposition.^7^ We did not, however, chose to include impulsivity as it is, in our view, not sufficiently specific to aggression. The lack of obvious differences in the results using either categorical or continuous approaches underscores our overall finding of no effect. Finally, for the analyses of continuous outcomes, in addition to the usual approach of combining the heterozygous with each of the two homozygous groups using dominant or recessive models, we also analyzed the additive genetic model, which is more consistent with polygenic inheritance.

However, there are some important limitations to this report. First, in order to utilize all available evidence, we initially employed a very inclusive definition of aggression and included dissimilar study populations ranging from university students to repeat offenders and patients in forensic psychiatry units. Thus, heterogeneity was expected in both the sample characteristics and the outcomes measured. We attempted to examine sources of this variation in predetermined sensitivity analyses by selecting specific phenotypes (differentiating aggression from anger) and more homogeneous samples with regard to ethnicity and age. These did not materially alter our overall findings, although at the 5% significance level, we reported some results. At most, these particular results are hypothesis generating, considering the number of tests undertaken. Further attempts to define more homogeneous groups measuring the same construct would result in fewer or single studies not suitable for meta-analysis. By pooling all available data together, it is possible that a ‘true' effect, if there is one, would emerge through the ‘noise'. The absence of significant associations indicates that any true effects would either be specific to certain populations or would be very small to be useful in clinical or judicial settings. On the other hand, it is equally possible that the ‘true' effects are diluted because of the heterogeneous sample sizes and outcomes, and this is the rationale for the detailed sensitivity analyses presented.

Furthermore, our data did not examine gene–environment interactions, and hence the impact of these genes may have been underestimated in this review. Doing so would have substantially limited the number of included studies and added an additional source of heterogeneity. However, most recent work in the field have examined such interactions, and there is some evidence for the interaction of *MAOA* genes and childhood maltreatment,^27, 54^ although this may be moderated by the degree of childhood trauma.^55^ A final limitation is that we did not report on some potentially important genes, including *NOS1* and *AVPR1A*,^9^ because of lack of sufficient evidence currently. The nonsignificant positive finding of an association with AR_(CAG)n suggest this polymorphism needs further examination. Development of a database, similar to the SZGene one (http://www.szgene.org/)^36^ would be helpful to the field to minimize publication bias, improve collaborations and allow for updated results to be analyzed.

It is notable that approximately half of the individual studies reported positive findings, which highlights that, among other things, findings from individual studies lacking sufficient power to detect anticipated small gene effects may have questionable validity. The use of many outcome measures without appropriate adjustment increases the chances of type I error. An alternative explanation is that true associations exist in specific populations and with the specific measures of aggression used. For example, one proposal is to divide aggression in two subtypes regulated by different neuronal pathways: the controlled–instrumental and the reactive–impulsive subtypes.^10^ However, the lack of consistency of the results limits the utility of the findings, which cannot be generalized in larger populations. Tests of publication bias were mostly not significant, although it should be noted that they had limited power to detect bias.^42, 56^

To our knowledge, this is the first systematic review of all the published genetic association studies of aggression and violence. Our study provides evidence that the candidate gene approach has not succeeded in identifying genes associated with these outcomes. This is consistent with recent observations in the field that candidate gene studies of human characteristics and complex diseases at large have failed to produce consistent and clinically useful findings.^57^ One explanation for this is that classic candidate gene studies are limited by *a priori* inferences on biological function of genes and tend to look at one variant that does not cover the full gene or relevant pathway. A hypothesis-free approach to the identification of new genes for aggression through genome-wide association studies or sequencing would be one obvious next step in order to identify genes for aggression. However, recent genome-wide association studies of personality traits or conduct disorder have only identified few markers of modest effect that are not yet clinically useful.^58, 59^ Possibly, larger samples to achieve adequate power are required and additional approaches, including the investigation of gene–environment interactions, the role of epigenetic regulation in aggression, determination of most informative markers and the better characterization of the phenotype, need to be explored further.^60^

As our review has identified no gene of major effect for aggression, any approach to use genetic markers for risk prediction, to mitigate criminal responsibility or to determine the treatment or management of specific individuals is questionable. The role of alternative research designs examining genetic associations to aggression and violence needs further examination.

## Figures and Tables

**Table 1:** Meta-analyses of categorical studies of genetic associations with aggression

*Gene*	N	*OR*	*95% CI*	P*-value*	p*(Q)*	I^*2*^*(%)*	*95% CI*
*HTR1B*	5	0.86	0.60–1.23	0.42	0.06	56	0–83
*5HTTLPR*	19	0.90	0.74–1.09	0.29	0	66	45–79
*5HTT-VNTR*	3	1.18	0.89–1.58	0.26	0.80	0	0–53
*BDNF*	4	0.86	0.65–1.13	0.27	0.20	35	0–77
*COMT*	19	0.84	0.63–1.02	0.08	0	64	41–78
*SLC6A3*^a^	3	1.19	0.91–1.56	0.20	0.92	0	0
*DRD4*^a^	4	1.40	0.87–2.26	0.17	0.09	53	0–85
*MAOA-F*	5	0.96	0.64–1.45	0.86	0.33	13	0–82
*MAOA-M*	17	0.82	0.65–1.05	0.13	0.01	50	13–71
*TPH1*	5	1.19	0.92–1.54	0.18	0.05	58	0–84
*AR (CAG)*	3	3.04	0.94–9.84	0.06	0	91	76–97
*DRD2*	3	1.30	0.99–1.71	0.06	0.26	25	0–92

Abbreviations: CI, confidence interval; *I*, *I*^2^ test for heterogeneity; *MAOA-F* and *MAOA-M*, *MAOA* in females and males, respectively; *N*, number of studies included; OR, pooled odds ratio; *p*(Q), *P*-value of Cochran's Q test for heterogeneity.

a Genotypic analysis of the dominant model for *SLC6A3* and *DRD4.*

**Table 2:** Meta-analyses of quantitative studies of genetic associations with aggression

*Gene*	*Model*	N	*ES*	*95% CI*	P*-value*	p*(Q)*	I^*2*^*(%)*	*95% CI*
*HTR2A*	Dom	3	−0.19	−0.39 to 0.01	0.06	0.12	52	0 to 86
	Rec	3	0.08	−0.15 to 0.32	0.49	0.10	56	0 to 87
	Add	3	0.24	−0.30 to 0.77	0.39	0.09	58	0 to 88
*5HTTLPR*	Dom	26	−0.08	−0.18 to 0.01	0.09	0.003	49	20 to 68
	Rec	31	−0.05	−0.14 to 0.05	0.32	0	60	41 to 73
	Add	26	0.17	−0.13 to 0.47	0.26	0	61	40 to 74
*BDNF*	Rec	3	−0.01	−0.12 to 0.09	0.79	0.50	0	0 to 85
*COMT*	Dom	21	−0.03	−0.15 to 0.09	0.64	0	58	32 to 74
	Rec	23	0.06	−0.04 to 0.17	0.24	0	58	33 to 73
	Add	21	−0.15	−0.49 to 0.19	0.39	0	60	35 to 75
*SLC6A3*	Dom	7	−0.06	−0.18 to 0.07	0.38	0.08	47	0 to 77
	Rec	3	0.29	−0.44 to 1.02	0.43	0.001	86	61 to 95
	Add	3	−0.07	−0.45 to 0.31	0.71	0.04	69	0 to 91
*DRD4*	Dom	14	0.04	−0.06 to 0.15	0.41	0.08	36	0 to 66
*MAOA-F*	Dom	7	0.001	−0.10 to 0.11	0.98	0.61	0	0 to 61
	Rec	6	0.13	−0.03 to 0.28	0.11	0.29	19	0 to 63
	Add	6	−0.11	−0.29 to 0.07	0.25	0.51	0	0 to 70
*MAOA-M*		16	0.08	−0.03 to 0.19	0.14	0.001	62	34 to 78
*TPH1*	Dom	11	0.08	−0.03 to 0.19	0.18	0.51	0	0 to 57
	Rec	12	0.05	−0.09 to 0.19	0.49	0.02	52	6 to 75
	Add	11	−0.12	−0.46 to 0.23	0.51	0.04	47	0 to 73
*AR (CAG)*		4	0.24	−0.50 to 0.99	0.52	0	92	84 to 96

Abbreviations: Add, additive model; CI, confidence interval; Dom, dominant model; ES, the effect size (Cohen's d for dominant and recessive, regression coefficient b for the additive model); *I*, *I*^2^ test for heterogeneity; *N*, number of studies included; *p*(Q), *P*-value of Cochran's Q test for heterogeneity; Rec, recessive model.

**Table 3:** Significant findings at the 5% level from subgroup analyses of genetic associations with aggression

*Gene*	*Subgroup*^a^	*Model*	N	*ES (95% CI)*	P-*value*	*Evidence*^b^
*Studies with categorical outcomes*						
* COMT*	Violence	Allelic	6	0.59 (0.38 to 0.92)	0.02	C
* MAOA-M*	Violence	Allelic	3	0.63 (0.42 to 1.24)	0.02	B
*TPH1*	Psychiatric patients	Allelic	4	1.31 (1.05 to 1.64)	0.01	B
*Studies with continuous outcomes*						
* 5HTTLPR*	Age >16 and <65	Dominant	22	−0.11 (−0.19 to −0.01)	0.02	C
* 5HTTLPR*	Substance users	Dominant	4	−0.35 (−0.62 to −0.09)	0.01	C
* COMT*	Caucasian	Recessive	16	0.14 (0.02 to 0.27)	0.02	C
* COMT*	Substance users	Recessive	4	0.31 (0.02 to 0.61)	0.04	B
* COMT*	Anger	Recessive	5	0.13 (0.02 to 0.24)	0.02	B
* COMT*	Caucasian	Additive	16	−0.37 (−0.71 to −0.02)	0.04	C
* COMT*	Substance users	Additive	4	−0.66 (−1.26 to −0.05)	0.03	B
* MAOA-F*	Aggressiveness	Recessive	4	0.24 (0.01 to 0.47)	0.04	B

ES (effect size) refers to odds ratio (OR) for categorical and b or Cohen's d for continuous outcomes (with 95% confidence interval (CI)).

a Subgroup analyses were performed by ethnicity, age of participants, sample characteristics and outcome measures.

b Assessment of cumulative evidence according to the HuGENet criteria. Details of grading scheme are in Supplementary Table 2.
