# Supplementary material for: Systematic meta-analyses and field synopsis of genetic association studies of violence and aggression
Source: Mol Psychiatry. 2013 Apr 2;19(4):471–7. doi: 10.1038/mp.2013.31 (PMC3965568; doi:10.1038/mp.2013.31)
Supplement: Supplementary Information [file mp201331x1.doc]

**Supplementary Material**

**Systematic Meta-Analyses and Field Synopsis of Genetic Association Studies of Violence and Aggression**

Evangelos Vassos, MD, David A Collier, PhD, Seena Fazel, MD

**Supplementary Material contents:**

**Supplementary Methods**

**Supplementary Table 1.** Detailed description of studies included in the review

**Supplementary Table 2.** Grading of the strength of cumulative evidence of meta-analyses with nominally positive findings according to the HuGENet criteria

**Supplementary Figure 1.** Forest Plots of meta-analyses of studies with categorical outcomes

**Supplementary Figure 2.** Forest Plots of meta-analyses of studies with continuous outcomes

**Supplementary References**

**Supplementary Methods**

**Measures of aggression**

In the categorical studies, cases were defined as individuals with a history of violence including homicide, “offenders”, “criminals”, persons with antisocial personality disorder, or people with history of aggression defined as a binary trait from the investigators of the original studies. Controls were usually taken from the general population. When the study reported two comparison groups, one with the same characteristics as the case group and the other a sample of the general population, we selected the comparator on the basis of being more similar to the cases (e.g. when aggressive alcoholics were compared with non-aggressive alcoholics and general population controls, we used the non-aggressive alcoholics as the preferred comparison group) and so that findings were more related to associations with violence rather than differences in the sample characteristics. Detailed description of the sample characteristics and definition of aggression is given in Supplementary Table 1.

In the studies examining quantitative traits, aggression was measured with various scales or questionnaires including the Buss-Durkee Hostility Inventory (BDHI),[^1^](#_ENREF_1) the Overt Aggression Scale (OAS),[^2^](#_ENREF_2) the Brown-Goodwin Lifetime History of Aggression (BGHA),[^3^](#_ENREF_3) the Behavioral Pathology in Alzheimer’s Disease (BEHAVE-AD),[^4^](#_ENREF_4) the Past Feelings and Acts of Violence Scale (PFAVS),[^5^](#_ENREF_5) the Neuropsychiatric Inventory (NPI),[^6^](#_ENREF_6) the Child Behavior Checklist (CBCL),[^7^](#_ENREF_7) the Revised Behavior Problem Checklist (RBPC),[^8^](#_ENREF_8) the Aggression Questionnaire (AQ),[^9^](#_ENREF_9) the Life History of Aggression (LHA),[^10^](#_ENREF_10) and custom-made composite indexes of aggression. Anger was measured in the majority of studies with State Trait Anger Expression Inventory (STAXI)[^11^](#_ENREF_11) (for our analyses we used the trait anger scale) or with the angry hostility facet of the NEO Personality Inventory.[^12^](#_ENREF_12)

When more than one scale was used by the investigators, we agreed in advance on the order of preference based on which scale more closely reflected the concept of externalized aggression. We favored physical to verbal aggression, and direct to indirect forms. This selection was blind to the direction of effect or the effect size of the association study. In the case of questionnaires with various subscales, we selected either the overall scale or the subscale that best reflected the previous concept. For example, in the case of BDHI we used the “assault/irritability” subscale when available, otherwise the total scale, and in OAS, we preferred the “physical aggression against other people” subscale.

**Data management**

The following data was extracted from each study: (i) allelic distribution and genotype frequency when available in studies with categorical outcomes, (ii) numbers, mean scores, and standard deviations (SD) of outcome by genotype in studies with continuous outcomes. Additional data extracted for each study was year of publication, study country, sex distribution, mean age, ethnic group, sample selection (general population, clinical, forensic or other specific sample), and the outcome measurement used. When genotypic distribution, means or standard deviations were not available for the preferred outcome measure, we contacted the authors. In total, we identified 37 relevant studies with missing data and we received tabular additional data from authors for 17 of those (46% response rate). This response rate was almost complete for studies published since 2008.

Articles reporting more than one genetic marker were included as independent samples for each marker. Similarly, publications that presented association results for males and females separately or with any other division of the total sample (e.g. different ethnic groups) were analyzed as independent samples.

Where associations of a particular polymorphism in the same or overlapping samples were reported in separately published articles, we selected the publication with the larger sample. If we were not certain about duplication of data, we contacted the corresponding authors of both articles, and, in the absence of a response, when duplication was probable, we took the conservative approach of excluding the smaller study.

When multiple identifiers were used for the same polymorphism, we used the most common nomenclature and recorded the NCBI reference rs number where available. We examined our dataset repeatedly to make sure that the order of alleles and genotypes was consistent across our list. In the case of complete linkage disequilibrium (LD) of two single nucleotide polymorphisms (SNPs) in the same gene (e.g. TPH1-A779C and TPH1-A218C), we pooled the study estimates together as if they referred to the same marker. As MAOA gene is x-linked, we performed separate meta-analyses for males and females. For variable number of tandem repeats (VNTR) polymorphisms with more than two variants (e.g. MAOA promoter or DRD4-ex3), we analyzed high versus low activity alleles based on the grouping of different number of repeats employed in each original study. Certain studies differed in the characterization of rare alleles (e.g. 5 repeats in MAOA 30bpVNTR have been included in either high or low activity markers). However as these variants are very rare, we assumed that they made little difference in the overall association and hence included them in the characterization presented in the original studies.

**Statistical analyses**

Studies with categorical and continuous outcomes of aggression were analyzed separately as the first employed case-control designs while the second examined aggression in a sample as a quantitative trait. For studies with binary outcomes, we tested allelic association (log-additive genetic model) and in studies with continuous outcomes we tested all 3 genetic models (dominant, recessive and additive) when sufficient data were available. In order for a polymorphism to be included in a meta-analysis, we required 3 or more separate samples to examine the association of this polymorphism with aggression as a quantitative trait or with violence as a binary outcome. The threshold of a minimum of 3 studies for the meta-analysis was selected so that there were at least two replication attempts of the original finding.[^13^](#_ENREF_13) However, all the primary studies that met the inclusion criteria are reported (Supplementary Table 1). Family-based association studies of related individuals were excluded. All statistical analyses were performed with STATA statistical software, release 10 (Stata Corp. 2007) and the R Project for Statistical Computing (www.r-project.org).

Evidence of deviation from Hardy-Weinberg equilibrium (HWE) was assessed by χ2 tests using the genotype frequencies of the sample analyzed in studies with continuous outcomes or the control group in the case-control approach of studies with binary outcomes. For the primary analyses, studies with HWE p-value <0.01 were excluded but we also performed post hoc analyses with the inclusion of studies that deviated from HWE. In the case of the X-linked MAOA genotype, when genotypes in females deviated from HWE, we also excluded males from the meta-analysis as this may have been an indication of problems with genotyping.

Studies that used both categorical and continuous outcome measures were included in the meta-analyses. For the categorical studies we calculated study-specific allelic odds ratios (ORs) and their 95% confidence intervals (CIs), with the exception of SLC6A3 where we calculated genotypic OR under the dominant model (9 repeats carriers vs 10 repeats homozygous) and DRD4-ex3 (presence of 7 repeats allele compared with all other genotypes). For continuous data, we computed Cohen's d effect sizes and their standard error (SE) from the reported means and SD. Whenever these data were not available, we computed d directly from the reported statistic (t or p value). We analyzed both dominant and recessive models by combining heterozygous with rare and common allele homozygous unless when the investigators consistently reported only combined genotypes (i.e. in the case of DRD4-ex3 or SLC6A3). To include analyses under the additive genetic model, which is more consistent with the polygenic theory and with the allelic approach taken in the case-control design, we estimated regression coefficients (β) and their SE from the given means and SD for the 3 genotypes (method available from the authors upon request). Power for the most commonly examined polymorphisms was calculated with the Genetic Power Calculator[^14^](#_ENREF_14) (http://pngu.mgh.harvard.edu/~purcell/gpc) using different ORs of 1.5, 1.2, and 1.1.

Initially, we calculated summary effect size and 95% CIs with the Der-Simonian and Laird random effects model,[^15^](#_ENREF_15) a more conservative approach to pooling data which utilizes weights that incorporate both the within and between study variance. This procedure was selected because, due to the broad definition of the aggression outcomes and variability of the samples, we expected large heterogeneity. Since a large number of analyses were performed, p-values of <0.05 are presented only as indicative of potential significance.

We estimated between-study heterogeneity by calculating the Cochran’s Q and the I^2^ statistic with its confidence intervals (CIs). The latter incorporates the percentage of variation across studies due to heterogeneity.[^18^](#_ENREF_18) As values of I^2^ > 50% are generally considered indicative of large heterogeneity, we repeated the analysis with fixed effect models using the Mantel-Haenszel weighting method when I^2^ were less than 50%. However, we favoured the random effects method because both Q and I^2^ metrics carry considerable uncertainty when few studies are included in a meta-analysis.

**Sensitivity analyses**

As we used a broad definition of aggression and samples were heterogeneous in relation to baseline characteristics, we performed the following post-hoc analyses: (1) excluding non-European and mixed ethnicity samples, (2) stratifying age groups (mean age<16, 16-65, and >65 years old), (3) separate analysis of samples from the general population, clinical samples with psychiatric disorder, offenders, and samples selected for substance use, (4) dividing the outcomes by severity to anger, general measures of aggressiveness including antisocial personality, history of violent acts, and criminal offending.

**Assessment of epidemiological credibility**

Evidence of publication bias was examined using Egger's, and Begg's tests in studies with continuous outcomes, and the modified version of Egger’s test[^23^](#_ENREF_23) in studies with a binary outcome measure. For meta-analyses with nominally significant findings, the strength of cumulative evidence was graded based on the Human Genome Epidemiology Network (HuGENet) guidelines[^24^](#_ENREF_24) (Supplementary Table 2).

**Supplementary Table 1.** Detailed description of studies included in the review.[^25-211^](#_ENREF_25)

| **Year** | **First author** | **Country** | **Ethnicity** | **Sample description** | **Mean age** | **Sex** | **M/F ratio** | **Measure** | **Instrument / method** | **Polymorphism** | **rs** | **N (total)** |
| --- | --- | --- | --- | --- | --- | --- | --- | --- | --- | --- | --- | --- |
| 1992 | Goldman | Finland | Caucasian | Alcoholics (& controls) | 29.2/30.1 | ? | N/A | violence in alcoholics | group comparison | DRD2-Taq1A | rs1800497 | 46 |
| 1994 | Comings | USA | Caucasian | Alcohol and drug users | ? | ? | N/A | Ever jailed for violence/ not | group comparison | DRD2-Taq1A | rs1800497 | 194 |
| 1995 | Vanyukov | USA | ? | Alcohol and drug users & control adolescents | 15.20 | both | 64/118 | Conduct disorder, aggression | group comparison MPQ, CBCL | MAOA Intron 2 VNTR | rs10548363 | 182 |
| 1997 | Strous | USA | mixed | S inpatients | 40.60 | both | 32/5 | aggression | records, history group comparison | COMT-Val158Met | rs4680 | 37 |
| 1998 | Sweet | USA | Caucasian | Probable Alzheimer's | 73.00 | both | 93/182 | physical aggression | CBRS group comparison | DRD1-48A/G | rs4532 | 193 |
| 1998 | Sweet | USA | Caucasian | Probable Alzheimer's | 73.00 | both | 93/182 | physical aggression | CBRS group comparison | DRD2-C311G | rs1801028 |  |
| 1998 | Sweet | USA | Caucasian | Probable Alzheimer's | 73.00 | both | 93/182 | physical aggression | CBRS group comparison | DRD3-*bal*I | rs6280 | 187 |
| 1998 | Sweet | USA | Caucasian | Probable Alzheimer's | 73.00 | both | 93/182 | physical aggression | CBRS group comparison | DRD4-ex3 48bp VNTR |  | 210 |
| 1998 | Lachman | USA | Caucasian | S patients | 42.80 | both | 34/21 | history of violence | group comparison (records, history) | COMT-Val158Met | rs4680 | 55 |
| 1998 | Lappalainen | Finland | Caucasian | Alcoholic criminals, family members, controls |  | both | 530/110 | APD or IED & alcoholism | group comparison (SCID to diagnose APD or IED) | HTR1B-G861C | rs6296 | 280 |
| 1998 | Lappalainen | USA | Indian-americans | Indian-american tribe (no selection) |  | both | 179/239 | APD & alcoholism | group comparison (with SADS) | HTR1B-G861C | rs6296 | 291 |
| 1998 | Nielsen | Finland | Caucasian | Alcoholic violent offenders, controls | 31.50 | M | N/A | history of violence | group comparison | TPH1-A779C | rs1799913 | 511 |
| 1998 | New | USA | Caucasian | PD patients | ? | M | N/A | impulsivity, aggression | BDHI, BIS | TPH1-A218C | rs1800532 | 21 |
| 1998 | Sander | Germany | Caucasian | APD vs non-APD alcoholics & controls | ? | both | ? | APD in alcoholics | group comparison, TPQ | 5HTTLPR | rs4795541 | 315 |
| 1998 | Mazzanti | Finland | Caucasian | Alcoholic criminal offenders, controls | ? | ? | ? | Offence & alcoholism | group comparison, TPQ | 5HTTLPR | rs4795541 | 397 |
| 1998 | Gelernter | USA | mixed | Substance abuse, pd, controls | 35.50 | both | 59/41 | (anger hostility) | NEO | 5HTTLPR | rs4795541 | 185 |
| 1999 | Rotondo | Finland | Caucasian | Violent offenders vs controls | 32.00 | M |  | history of violence, arson | group comparison | TPH -6526 |  | 320 |
| 1999 | Huang | USA | ? | brain tissue normal & suicide | 41.70 | both | 134/44 | h of violence, suicide | records, history group comparison | HTR1B-G861C | rs6296 | 78 |
| 1999 | Manuck | USA | mixed | normal | 45.70 | both | 124/127 | aggression, anger | LHA, BDHI, STAXI | TPH1-A218C | rs1800532 | 251 |
| 1999 | Kotler | Israel | Caucasian | violent S vs non-violent S (& controls) | 42.20 | both | 58/34 | homicide | group comparison | COMT-Val158Met | rs4680 | 92 |
| 1999 | Kotler | Israel | Caucasian | violent S vs non-violent S (& controls) | 42.20 | both | 58/34 | homicide | group comparison | DRD4-ex3 48bp VNTR |  | 91 |
| 1999 | Kotler | Israel | Caucasian | violent S vs non-violent S (& controls) | 42.20 | both | 58/34 | homicide | group comparison | 5HTTLPR | rs4795541 | 82 |
| 1999 | Lappalainen | Finland | Caucasian | violent alcoholics/controls | 38.00 | M | N/A | APD & alcoholism | group comparison | HTR2C-Cys23Ser | rs6318 | 436 |
| 1999 | Hallikainen | Finland | Caucasian | violent vs non-violent alcoholics (& controls) |  | M | N/A | violent offence & alcoholism | group comparison | 5HTTLPR | rs4795541 | 165 |
| 1999 | Samochowiec | Germany | Caucasian | APD vs non-APD alcoholics & controls | 43.80 | M | N/A | APD in alcoholics | group comparison | MAOA promoter 30bpVNTR |  | 303 |
| 1999 | Wei | UK | Caucasian | S patients |  | both | 29/20 | Violent behavior | group comparison | COMT (other) | [various SNPs] | 49 |
| 1999 | Ishiguro | Japan | Asian | antisocial alcoholics (& non-antisocial) /controls | 52.20 | both | 153/13 | Antisocial behavior & alcoholism | group comparison | 5HTTLPR | rs4795541 | 166 |
| 2000 | Nolan | USA | Caucasian | S patients | ? | M | N/A | history of violence | group comparison | TPH1-A779C | rs1799913 | 55 |
| 2000 | Nolan | USA | Caucasian | S patients | ? | M | N/A | history of violence | group comparison | 5HTTLPR | rs4795541 | 56 |
| 2000 | Nolan | USA | Caucasian | S patients | ? | M | N/A | history of violence | group comparison | MAOA promoter 30bpVNTR |  | 54 |
| 2000 | Comings | USA | Caucasian | 2 samples: students, parents of twins |  | both | 40/60 | irritability hostility | BADD, BDHI, MPQ | ADRA2A-promoter | rs1800544 | 123 |
| 2000 | Manuck | USA | Caucasian | normal | 45.20 | M | N/A | aggression impulsivity | LHA, BIS, BDHI | MAOA promoter 30bpVNTR |  | 110 |
| 2000 | Persson | Sweden | Caucasian | normal | 41.00 | both | 109/96 | anger hostility | NEO | TH (TCAT)n VNTR |  | 58 |
| 2000 | Greenberg | USA | mixed | normal |  | both |  | anger hostility | NEO | 5HTTLPR | rs4795541 | 902 |
| 2000 | Damberg | Sweden | Caucasian | normal | ? | both | 64/73 | indirect aggression | KSP | AP-2beta |  | 137 |
| 2000 | Hallikainen | Finland | Caucasian | violent alcoholics vs controls | 30.4/54.6 | M | N/A | violent offence & alcoholism | group comparison | COMT-Val158Met | rs4680 | 329 |
| 2000 | Evans | UK | ? | Suicide attempters | 30.16 | both | 174/182 | impulsiveness | IVE impuls. Quest, STAXI | TPH1-A779C | rs1799913 | 350 |
| 2000 | Evans | UK | ? | Suicide attempters | 30.16 | both | 174/182 | impulsiveness | IVE impuls. Quest, STAXI | HTR2C-Cys23Ser | rs6318 |  |
| 2001 | Sweet | USA | ? | Probable Alzheimer's | 75.40 | both | 36/64 | aggression | EBEHAVE, ERS group comparison | 5HTTLPR | rs4795541 | 332 |
| 2001 | Holmes | UK | Caucasian | Probable Alzheimer's | 83.50 | both | 23/77 | aggression | CAMDEX, group comparison | DRD1-48A/G | rs4532 | 105 |
| 2001 | Holmes | UK | Caucasian | Probable Alzheimer's | 83.50 | both | 23/77 | aggression | CAMDEX, group comparison | DRD3-*bal*I | rs6280 |  |
| 2001 | New | USA | mixed | PD patients | 38.10 | both | 86/59 | impulsive aggression | BDHI | HTR1B-G861C | rs6296 | 145 |
| 2001 | Zalsman | Israel | Caucasian | adolescents patients suicide attempters | 17.40 | both | 40/48 | violence | PFAVS, IS | TPH1-A218C | rs1800532 | 88 |
| 2001 | Jonsson | Sweden | Caucasian | healthy subjects | 42.90 | both | 185/152 | aggression | KSP | AR_(CAG)n | rs72181988 | 335 |
| 2001 | Reist | USA | ? | alcoholics and controls | 40.80 | M | N/A | aggression, hostility | BIS, BGLHA, BDHI | 5HTTLPR | rs4795541 | 27 |
| 2001 | Zalsman | Israel | Caucasian | adolescents patients suicide attempters | 18.90 | both | 16/32 | violence, impulsivity | PFAVS, IS | 5HTTLPR | rs4795541 | 46 |
| 2001 | Preuss | Germany | Caucasian | alcoholics | 42.00 | both | 107/28 | impulsivity | BIS | HTR2A-1438A/G | rs6311 | 135 |
| 2001 | Liou | Taiwan | Asian | S patients | 37.80 | both | 94/104 | h of violence | group comparison | COMT-Val158Met | rs4680 | 198 |
| 2001 | Parsian | USA | Caucasian | APD vs non-APD alcoholics & controls |  | both | 102/31 | APD in alcoholics | group comparison | 5HTTLPR | rs4795541 | 133 |
| 2001 | Matsushita | Japan | Caucasian | alcoholics (& controls) | 50.50 | M | N/A | violence while intoxicated | group comparison | 5HTTLPR | rs4795541 | 369 |
| 2001 | Twitchell | USA | Caucasian | children of alcoholics | 10.90 | both | 45/17 | Aggressive behavior | CBCL | 5HTTLPR | rs4795541 | 62 |
| 2001 | Jorm | Australia | Caucasian | children & adolescents | 16.00 | both | ? | Conduct disorder, aggression | RBPC, Rutter PBQ | SLC6A3-40bpVNTR |  | 680 |
| 2002 | Manuck | USA | mixed | normal | 44.30 | M | N/A | aggression | LHA, (also BDHI, BIS) | MAOA promoter 30bpVNTR |  | 58 |
| 2002 | Rujescu | Germany | Caucasian | healthy/suicide attempters | 44.70 | both | 93/147 | hostility, anger | BDHI, STAXI | TPH1-A779C | rs1799913 | 214 |
| 2002 | Avramopoul | Greece | Caucasian | healthy | 21.00 | M | N/A | aggression | AQ | COMT-Val158Met | rs4680 | 334 |
| 2002 | Caspi | New Zealand | Caucasian | young w or w/out maltreatment | 26.00 | M | N/A | violence | group comparison, convictions, DTVS, APD | MAOA promoter 30bpVNTR |  | 442 |
| 2002 | Patkar | USA | Black | cocaine users (& controls) | 36.60 | both | 75/30 | aggression, impulsivity | BDHI, SSS, BIS, ASI | 5HTTLPR | rs4795541 | 105 |
| 2002 | Patkar | USA | Black | cocaine users (& controls) | 36.60 | both | 75/30 | aggression, impulsivity | BDHI, SSS, BIS, ASI | 5HTT-VNTR |  | 103 |
| 2002 | Staner | Luxembourg | Caucasian | Impulsive inpatients non-psychotic | 38.80 | both | 34/20 | hostility | BIS, BDHI, SADS | TPH1-A218C | rs1800532 | 54 |
| 2002 | Staner | Luxembourg | Caucasian | Healthy controls | 35.20 | both | 11/16 | hostility | BIS, BDHI, SADS | TPH1-A218C | rs1800532 | 27 |
| 2002 | Saito | Finland | Caucasian | violent vs non-violent alcoholics (& controls) |  | M | N/A | violent offence & alcoholism | group comparison | MAOA promoter 30bpVNTR |  | 172 |
| 2002 | Hill | USA | Caucasian | Antisocial alcoholic probands & parents |  | both | 26/11 | antisocial behavior | ABC | HTTLPR, TPH, HTR1b, HTR2A, HTR2C, SLC6A4, MAO-A |  | 37 |
| 2002 | Stoltenberg | USA | Caucasian | Antisocial vs non-antisocial alcoholics | ? | M | N/A | antisocial behavior | group comparison | 5HTTLPR | rs4795541 | 33 |
| 2002 | Young | USA | ? | Children | 7.00 | both | ? | Externalizing behavior | CBCL | SLC6A3-40bpVNTR |  | 790 |
| 2002 | Kranzler | USA | Caucasian | substance dependence +/- ASPD (& controls) |  |  |  | APD | group comparison | HTR1B-G861C | rs6296 | 278 |
| 2002 | Kranzler | USA | Black | substance dependence +/- ASPD (& controls) |  |  |  | APD | group comparison | HTR1B-G861C | rs6296 | 69 |
| 2002 | Comings | USA | Caucasian | Substance abuse, pd, controls |  | M |  | Aggression | BDHI | AR_(GGC)n |  | 121 |
| 2003 | Berggård | Sweden | Caucasian | criminals/controls | 35.00 | M | N/A | violence | group comparison | HTR2A-1438A/G | rs6311 | 317 |
| 2003 | Rujescu | Germany | Caucasian | suicide attempters & controls | 44.00 | both | 202/275 | aggression | STAXI, group comparison | COMT-Val158Met | rs4680 | 411 |
| 2003 | Strous | Israel | Caucasian | S patients |  | M | N/A | aggression | LHA, group comparison | COMT-Val158Met | rs4680 | 94 |
| 2003 | Strous | Israel | Caucasian | S patients |  | F | N/A | aggression | LHA, group comparison | COMT-Val158Met | rs4680 | 28 |
| 2003 | Strous | Israel | Caucasian | S patients |  | M | N/A | aggression | LHA, group comparison | MAOA promoter 30bpVNTR |  | 95 |
| 2003 | Strous | Israel | Caucasian | S patients |  | F | N/A | aggression | LHA, group comparison | MAOA promoter 30bpVNTR |  | 29 |
| 2003 | Retz | Germany | Caucasian | offenders, various diagnoses | 34.70 | M | N/A | violence, impulsiveness | group comparison EIQ, WURS | DRD3-*bal*I | rs6280 | 146 |
| 2003 | Westberg | Sweden | Caucasian | volunteers | 42.00 | F | N/A | aggression, irritability | KSP | ER-alpha VNTR |  | 172 |
| 2003 | Koller | Germany | Caucasian | alcoholics (& controls) | 41.80 | M | N/A | aggression, irritability | BIS, BGLHA, BDHI, group comparison | MAOA promoter 30bpVNTR |  | 169 |
| 2003 | Lu | Taiwan | Asian | APD jailed/ controls (& non-APD jailed) | 30.00 | M | N/A | APD jailed ± alcoholism | group comparison | MAOA promoter 30bpVNTR |  | 168 |
| 2003 | Cadoret | USA | ? | Adoptees | ? | both | 37/50 | aggressivity | Behavior scales | 5HTTLPR | rs4795541 | 87 |
| 2003 | Sen | USA | Caucasian | Hypertensives | 33.00 | both | 173/268 | Hostility | NEO | BDNF-Val66Met | rs6265 | 439 |
| 2004 | Han | Korea | Asian | S patients (& controls) |  | M | N/A | aggression | OAS | COMT-Val158Met | rs4680 | 168 |
| 2004 | Han | Korea | Asian | S patients (& controls) |  | M | N/A | aggression | OAS | 5HTTLPR | rs4795541 | 168 |
| 2004 | Assal | USA | mixed | Alzheimer's | 76.70 | both | 37/59 | agitation, aggression | NPI | 5HTTLPR | rs4795541 | 96 |
| 2004 | Assal | USA | mixed | Alzheimer's | 76.70 | both | 37/59 | agitation, aggression | NPI | HTR2A-T102C | rs6313 | 96 |
| 2004 | Assal | USA | mixed | Alzheimer's | 76.70 | both | 37/59 | agitation, aggression | NPI | HTR2C-Cys23Ser | rs6318 | 96 |
| 2004 | Assal | USA | mixed | Alzheimer's | 76.70 | both | 37/59 | agitation, aggression | NPI | 5HTT-VNTR |  | 96 |
| 2004 | Retz | Germany | Caucasian | forensic referrals (violent/no violent) | 34.70 | M | N/A | violence | EIQ, WURS, group comparison | 5HTTLPR | rs4795541 | 153 |
| 2004 | Lam | China | Asian | Alzheimer's | 77.40 | both |  | agitation, aggression | NPI group comparison | HTR2A-T102C | rs6313 | 87 |
| 2004 | Craig | UK | ? | Alzheimer's | 78.00 | both | 139/257 | agitation, aggression | NPI group comparison | TPH1-A218C | rs1800532 | 396 |
| 2004 | Huang | USA | mixed | mood disorders | 37.60 | M | N/A | aggression | BIS, BGLHA, BDHI | MAOA promoter 30bpVNTR |  | 272 |
| 2004 | Huang | USA | mixed | mood disorders | 37.60 | F | N/A | aggression | BIS, BGLHA, BDHI | MAOA promoter 30bpVNTR |  | 398 |
| 2004 | Volavka | USA | mixed | S patients | 40.80 | both | 48/12 | hostility | PANSS hostility item | COMT-Val158Met | rs4680 | 60 |
| 2004 | Gerra | Italy | Caucasian | heroin users (&controls) | 33.70 | M | N/A | aggression | BDHI, forensic examination | MAOA promoter 30bpVNTR |  | 104 |
| 2004 | Gerra | Italy | Caucasian | heroin users | 34.60 | M | N/A | aggression | BDHI, forensic examination | 5HTTLPR | rs4795541 | 101 |
| 2004 | Gerra | Italy | Caucasian | (controls) | 35.80 | M | N/A | aggression | BDHI, forensic examination | 5HTTLPR | rs4795541 | 101 |
| 2004 | Koen | South Africa | mixed | S patients |  | M | N/A | violence towards self or others, or violent threats | group comparison CABS, PANSS, OAS | COMT-Val158Met | rs4680 | 63 |
| 2004 | Koen | South Africa | mixed | S patients |  | M | N/A | violence | group comparison CABS, PANSS, OAS | MAOA promoter 30bpVNTR |  | 63 |
| 2004 | Liao | Taiwan | Asian | violent criminals/controls | 34.70 | M | N/A | violence | group comparison | 5HTTLPR | rs4795541 | 246 |
| 2004 | Matsushita | Japan | Asian | alcoholics (& controls) | 49.90 | M | N/A | violence while intoxicated | group comparison | BDNF-Val66Met | rs6265 | 377 |
| 2004 | Foley | USA | Caucasian | Twins | 12.23 | M | N/A | conduct disorder | CD group comparison | MAOA promoter 30bpVNTR |  | 514 |
| 2004 | Zalsman | Israel | Caucasian | suicide attempters | 17.70 | both | 29/40 | violence | PFAVS, IS | DRD4-ex3 48bp VNTR |  | 67 |
| 2004 | Zammit | UK | Caucasian | S patients (& controls) |  | both | 136/44 | aggression | overt aggression scale | COMT-Val158Met | rs4680 | 326 |
| 2004 | Zammit | UK | Caucasian | S patients (& controls) |  | M | N/A | aggression | overt aggression scale | MAOA promoter 30bpVNTR |  | 120 |
| 2004 | Zammit | UK | Caucasian | S patients (& controls) |  | F | N/A | aggression | overt aggression scale | MAOA promoter 30bpVNTR |  | 42 |
| 2004 | Baca-Garcia | Spain | Caucasian | suicide attempters only | 36.80 | M | N/A | Aggressive behavior | BGLAS, BIS, group comparison | 5HTTLPR | rs4795541 | 63 |
| 2004 | Baca-Garcia | Spain | Caucasian | suicide attempters only | 36.80 | F | N/A | Aggressive behavior | BGLAS, BIS, group comparison | 5HTTLPR | rs4795541 | 139 |
| 2004 | Baca-Garcia | Spain | Caucasian | controls only |  | M | N/A | Aggressive behavior | BGLAS, BIS, group comparison | 5HTTLPR | rs4795541 | 87 |
| 2004 | Baca-Garcia | Spain | Caucasian | controls only |  | F | N/A | Aggressive behavior | BGLAS, BIS, group comparison | 5HTTLPR | rs4795541 | 77 |
| 2005 | Gerra | Italy | Caucasian | heroin users (&controls) | 33.70 | M | N/A | aggression | BDHI, forensic examination | SLC6A3-40bpVNTR |  | 104 |
| 2005 | Chen |  |  | pathol aggressive adolescents/super controls/controls |  | ? |  | aggression violence | group comparison? | SLC6A3-40bpVNTR |  | 41 |
| 2005 | Chen |  |  | pathol aggressive adolescents/super controls/controls |  | ? |  | aggression violence | group comparison? | DRD2-Taq1A | rs1800497 | 41 |
| 2005 | Tsai | Taiwan | Asian | violent criminals/controls | 34.70 | M | N/A | violence | history, MINI, group comparison | BDNF-Val66Met | rs6265 | 283 |
| 2005 | Jacob | Germany | Caucasian | PD/controls |  | both |  | PD cluster, aggressiveness | SCID II, NEO-PI, TPQ | MAOA promoter 30bpVNTR |  | 847 |
| 2005 | Gerra | Italy | Caucasian | students (14-19) +/-using drugs | 16.74 | both | 129/87 | personality, aggressiveness | BDHI, TPQ | 5HTTLPR | rs4795541 | 216 |
| 2005 | Reuter | Germany | Caucasian | normal | 25.43 | both | 125/127 | indirect hostility | BDHI | TPH1-A779C | rs1799913 | 252 |
| 2005 | Kweon | Korea | Asian | violent vs non-violent alcoholics (& controls) | 45.70 | M | N/A | violence | group comparison | COMT-Val158Met | rs4680 | 97 |
| 2005 | Kweon | Korea | Asian | violent vs non-violent alcoholics (& controls) | 45.70 | M | N/A | violence | group comparison | 5HTTLPR | rs4795541 | 145 |
| 2005 | Haberstick | USA | Caucasian | young w or w/out maltreatment | ? | M | N/A | conduct problems | Lifetime conduct problems | MAOA promoter 30bpVNTR |  | 774 |
| 2005 | Ha | Korea | Asian | Alzheimer's | 74.90 | both | 27/38 | aggression | BEHAVE-AD group comparison | 5HTTLPR | rs4795541 | 65 |
| 2006 | de Young | Canada | mixed | children | 10.00 | M | N/A | externalizing behavior | informant rating scales | DRD4-ex3 48bp VNTR |  | 48 |
| 2006 | de Young | Canada | mixed | adults with ADHD | 35.17 | M | N/A | externalizing behavior | informant rating scales | DRD4-ex3 48bp VNTR |  | 42 |
| 2006 | de Young | Canada | mixed | boys | 17.00 | M | N/A | externalizing behavior | informant rating scales | DRD4-ex3 48bp VNTR |  | 87 |
| 2006 | Eisenberger | USA | mixed | community sample | 20.60 | both | 13/19 | aggression | BSI, Spielberger anger trait | MAOA promoter 30bpVNTR |  | 32 |
| 2006 | Sakai | USA | mixed | adolescents w CD & aggression /controls | 15.50 | both | 252/145 | conduct disorder with aggression | CIDI, DISC, group comparison | 5HTTLPR | rs4795541 | 339 |
| 2006 | Giegling | Germany | Caucasian | suicide attempters & controls together | 40.00 | both | 173/243 | anger aggression | STAXI, BDHI | HTR2A-1438A/G | rs6311 | 416 |
| 2006 | Widom | USA | mixed | adults who had been abused as children and controls | 41.00 | both | 268/141 | violence antisocial behavior | composite index: criminal history, self-report, DIS | MAOA promoter 30bpVNTR |  | 409 |
| 2006 | Beitchman | Canada | ? | aggressive children/controls | 9.54 | both | 70/12 | aggression | group comparison CBC, TRF | 5HTTLPR | rs4795541 | 318 |
| 2006 | Beitchman | Canada | ? | aggressive children/controls | 9.54 | both | 70/12 | aggression | group comparison CBC, TRF | 5HTT-VNTR |  | 146 |
| 2006 | Han | Korea | Asian | 1st onset S (&controls) | 27.00 | M | N/A | aggression | overt aggression scale | COMT-Val158Met | rs4680 | 132 |
| 2006 | Verona | USA | mixed | students | 21.00 | both | 55/56 | aggression | laboratory experimet | 5HTTLPR | rs4795541 | 111 |
| 2006 | Cheng | Taiwan | Asian | violent criminals /controls |  | M | N/A | violent criminal activity | group comparison | AR_(CAG)n | rs72181988 | 254 |
| 2006 | Nilsson | Sweden | Caucasian | adolescents | 19.00 | M | N/A | criminal activity | criminality index | MAOA promoter 30bpVNTR |  | 78 |
| 2006 | Pritchard | UK | Caucasian | Alzheimer's | 74.40 | both | 173/220 | agitation, aggression | NPI, group comparison | HTR2A-T102C | rs6313 | 393 |
| 2006 | Pritchard | UK | Caucasian | Alzheimer's | 74.40 | both | 173/220 | agitation, aggression | NPI, group comparison | HTR2C-Cys23Ser | rs6318 | 393 |
| 2006 | Haberstick | USA | Caucasian | School children from general population | 9.50 | both | 358/374 | Aggressive behavior | Parent and teacher rating scales | 5HTTLPR | rs4795541 | 732 |
| 2006 | Sengupta | Canada | mixed | Children w ADHD | 9.00 | both | 167/24 | conduct disorder | CD symptom score | COMT-Val158Met | rs4680 | 191 |
| 2006 | Kim-Cohen | UK | Caucasian | Twins | 7.00 | M | N/A | Antisocial behavior | Antisocial behavior scale | MAOA promoter 30bpVNTR |  | 975 |
| 2006 | Huizinga | USA | Caucasian | Adolescents |  | M | N/A | Violence | composite index: criminal history, self-report, CD | MAOA promoter 30bpVNTR |  | 277 |
| 2007 | Guerin | Canada | mixed | aggressive children/ adult controls | ? | both | ? | aggression | DISC, group comparison | BDNF-Val66Met | rs6265 | 170 |
| 2007 | Pritchard | UK | Caucasian | Probable Alzheimer's | 74.40 | both | 44/56% | aggression irritability | NPI group comparison | ApoE (various) |  | 388 |
| 2007 | Pritchard | UK | Caucasian | Probable Alzheimer's | 74.40 | both | 44/56% | aggression irritability | NPI group comparison | 5HTTLPR | rs4795541 | 367 |
| 2007 | Pritchard | UK | Caucasian | Probable Alzheimer's | 74.40 | both | 44/56% | aggression irritability | NPI group comparison | 5HTT-VNTR |  | 257 |
| 2007 | Guo | USA | mixed | adolescents (sibs & twins!) |  | both | 1/1 | violent delinquency | delinquency scales | SLC6A3-40bpVNTR |  | 2530 |
| 2007 | Guo | USA | mixed | adolescents (sibs & twins!) |  | both | 1/1 | violent delinquency | delinquency scales | DRD2-Taq1A | rs1800497 | 2525 |
| 2007 | Sjoberg | Sweden | Caucasian | adolescents | 17.00 | F | N/A | criminal activity | criminality index | MAOA promoter 30bpVNTR |  | 115 |
| 2007 | Perlis | USA | Caucasian | depressed | 39.20 | both | 42/52 | anger expression | STAXI | CREB1 (various) | [6 SNPs] | 94 |
| 2007 | Wasserman | Ukraine | Caucasian | parents of suicidal probands, controls | 48.00 | both | 49/51 | anger-hostility | NEO | TBX19 | [various SNPs] | 744 |
| 2007 | Sakai | USA | mixed | adolescents w CD & aggression /controls | 16.09 | both | 48/52 | conduct disorder with aggression | CIDI, DISC, group comparison | 5HTTLPR | rs4795541 | 1736 |
| 2007 | Reif | Germany | Caucasian | forensic referrals (violent/no violent) | 34.10 | M | N/A | violence | group comparison | MAOA promoter 30bpVNTR |  | 184 |
| 2007 | Reif | Germany | Caucasian | forensic referrals (violent/no violent) | 34.10 | M | N/A | violence | group comparison | 5HTTLPR | rs4795541 | 184 |
| 2007 | Reif | Germany | Caucasian | forensic referrals (violent/no violent) | 34.10 | M | N/A | violence | group comparison | SLC6A3-40bpVNTR |  | 184 |
| 2007 | Gietl | Germany | Caucasian | Suicide attempters, & controls | ? | both | 198/283 | anger aggression | STAXI, FAF | ABCG1 | [5 SNPs] | 414 |
| 2007 | Serretti | Germany | Caucasian | Suicide attempters, & controls | ? | both |  | anger aggression | STAXI, FAF | HTR2C-Cys23Ser | rs6318 | 404 |
| 2007 | Serretti | Germany | Caucasian | Suicide attempters, & controls | ? | both |  | anger aggression | STAXI, FAF | HTR1A | rs6295 | 404 |
| 2007 | Giegling | Germany | Caucasian | Suicide attempters, & controls | ? | both | 198/283 | anger aggression | STAXI, FAF | TACR1 | [4 SNPs] |  |
| 2007 | Zouk | Canada | Caucasian | suicide completers (& controls) | 38.00 | both | 274/64 | Aggression (proxy interview) | BDHI, BIS, BGHA | HTR1B-G861C | rs6296 | 322 |
| 2007 | Frazzetto | Italy | Caucasian | psychiatric outpatients, controls | 32.18 | M | N/A | aggression | aggression questionnaire | MAOA promoter 30bpVNTR |  | 82 |
| 2007 | Frazzetto | Italy | Caucasian | psychiatric outpatients, controls | 32.18 | F | N/A | aggression | aggression questionnaire | MAOA promoter 30bpVNTR |  | 153 |
| 2007 | Silva | Chile | mixed | Borderline PD | ? | both | 13/36 | aggression | OAS | 5HTTLPR | rs4795541 | 49 |
| 2007 | Fresan | Mexico | ? | schizophrenic patients | 30.50 | both | 22/49 | aggression | group comparison & OAS | DRD4-ex3 48bp VNTR |  | 71 |
| 2007 | Fresan | Mexico | ? | schizophrenic patients | 30.50 | M | N/A | aggression | group comparison & OAS | MAOA promoter 30bpVNTR |  | 49 |
| 2007 | Fresan | Mexico | ? | schizophrenic patients | 30.50 | F | N/A | aggression | group comparison & OAS | MAOA promoter 30bpVNTR |  | 22 |
| 2007 | Yang | Korea | Asian | Students | 22.80 | F | N/A | anger | STAXI | MAOA promoter 30bpVNTR |  | 211 |
| 2007 | Flory | USA | Caucasian | Personality disorders | ? | both | 76/36 | Hostility | BDHI | COMT-Val158Met | rs4680 | 112 |
| 2007 | Nobile | Italy | Caucasian | general population preadolescents | 12.11 | both | 309/298 | Aggressive behavior | CBCL | 5HTTLPR | rs4795541 | 589 |
| 2007 | Nobile | Italy | Caucasian | general population preadolescents | 12.11 | both | 309/298 | Aggressive behavior | CBCL | DRD4-ex3 48bp VNTR |  | 589 |
| 2007 | Gerra | Italy | Caucasian | cocaine users (& controls) | 22.60 | M | N/A | aggressiveness | BDHI | 5HTTLPR | rs4795541 | 96 |
| 2008 | Schulz-Heik | USA | mixed | adolescents w CD & substance /controls | 15.80 | both | ? | conduct disorder | group comparison | SLC6A3-40bpVNTR |  | 303 |
| 2008 | Keltikangas | Finland | Caucasian | healthy population | 28.00 | both | 313/416 | hostility | anger, cynicism, paranoia scales | HTR1A | rs6295 | 729 |
| 2008 | Keltikangas | Finland | Caucasian | healthy population | 28.00 | both | 313/416 | hostility | anger, cynicism, paranoia scales | HTR2A-T102C | rs6313 | 729 |
| 2008 | Rujescu | Germany | Caucasian | Suicide attempters, & controls | ? | both | 198/283 | anger aggression | STAXI, FAF | NOS1 | [4 SNPs] | 571 |
| 2008 | Kang | Korea | Asian | Students | 22.50 | M | N/A | anger | STAXI, TFS | DRD4-ex3 48bp VNTR |  | 184 |
| 2008 | Kang | Korea | Asian | Students | 22.50 | F | N/A | anger | STAXI, TFS | DRD4-ex3 48bp VNTR |  | 124 |
| 2008 | Kang | Korea | Asian | Students | 22.50 | M | N/A | anger | STAXI, TFS | COMT-Val158Met | rs4680 | 184 |
| 2008 | Kang | Korea | Asian | Students | 22.50 | F | N/A | anger | STAXI, TFS | COMT-Val158Met | rs4680 | 124 |
| 2008 | Guo | USA | mixed | adolescents (sibs & twins!) | 15.60 | M | N/A | violent delinquency | delinquency scales | MAOA promoter 30bpVNTR |  | 1200 |
| 2008 | Guo | USA | mixed | adolescents (sibs & twins!) | 15.50 | F | N/A | violent delinquency | delinquency scales | MAOA promoter 30bpVNTR |  | 1324 |
| 2008 | Caspi | UK | Caucasian | ADHD children (Cardiff) | 9.20 | both | 214/27 | conduct problems | CAPA | COMT-Val158Met | rs4680 | 241 |
| 2008 | Caspi | UK | Caucasian | ADHD children (Cardiff) | 9.20 | both | 214/27 | conduct problems | CAPA | SLC6A3-40bpVNTR |  | 186 |
| 2008 | Caspi | UK | Caucasian | ADHD children (Cardiff) | 9.20 | both | 214/27 | conduct problems | CAPA | DRD4-ex3 48bp VNTR |  | 184 |
| 2008 | Caspi | UK | Caucasian | ADHD children (E-risk) | 7.00 | both |  | Aggressive behavior | Child behavior checklist | COMT-Val158Met | rs4680 | 185 |
| 2008 | Caspi | UK | Caucasian | controls (E-risk) | 7.00 | both |  | Aggressive behavior | Child behavior checklist | COMT-Val158Met | rs4680 | 1839 |
| 2008 | Caspi | UK | Caucasian | ADHD children (E-risk) | 7.00 | both |  | Aggressive behavior | Child behavior checklist | SLC6A3-40bpVNTR |  | 180 |
| 2008 | Caspi | UK | Caucasian | controls (E-risk) | 7.00 | both |  | Aggressive behavior | Child behavior checklist | SLC6A3-40bpVNTR |  | 1788 |
| 2008 | Caspi | UK | Caucasian | ADHD children (E-risk) | 7.00 | both |  | Aggressive behavior | Child behavior checklist | DRD4-ex3 48bp VNTR |  | 184 |
| 2008 | Caspi | UK | Caucasian | controls (E-risk) | 7.00 | both |  | Aggressive behavior | Child behavior checklist | DRD4-ex3 48bp VNTR |  | 1803 |
| 2008 | Caspi | New Zealand | Caucasian | ADHD children (Dunedin) | 26.00 | both |  | Antisocial behavior, adult court convictions | Antisocial composite index, group comparison | COMT-Val158Met | rs4680 | 49 |
| 2008 | Caspi | New Zealand | Caucasian | controls (Dunedin) | 26.00 | both |  | Antisocial behavior, adult court convictions | Antisocial composite index, group comparison | COMT-Val158Met | rs4680 | 787 |
| 2008 | Caspi | New Zealand | Caucasian | ADHD children (Dunedin) | 26.00 | both |  | Antisocial behavior, adult court convictions | Antisocial composite index, group comparison | SLC6A3-40bpVNTR |  | 48 |
| 2008 | Caspi | New Zealand | Caucasian | controls (Dunedin) | 26.00 | both |  | Antisocial behavior, adult court convictions | Antisocial composite index, group comparison | SLC6A3-40bpVNTR |  | 727 |
| 2008 | Caspi | New Zealand | Caucasian | ADHD children (Dunedin) | 26.00 | both |  | Antisocial behavior, adult court convictions | Antisocial composite index, group comparison | DRD4-ex3 48bp VNTR |  | 49 |
| 2008 | Caspi | New Zealand | Caucasian | controls (Dunedin) | 26.00 | both |  | Antisocial behavior, adult court convictions | Antisocial composite index, group comparison | DRD4-ex3 48bp VNTR |  | 799 |
| 2008 | Rajender | India | Caucasian | criminals/controls |  | M | N/A | serious offence (rape, murder) | group comparison | AR_(CAG)n | rs72181988 | 645 |
| 2008 | Giegling | Germany | Caucasian | Suicide attempters, & controls | 43.20 | both | 196/283 | anger aggression | STAXI, FAF | ESR1 | [6 SNPs] | ? |
| 2008 | Kulikova | Russia | Caucasian | Young women | 17.00 | F | N/A | Aggressive behavior | Buss-Darky? | COMT-Val158Met | rs4680 | 114 |
| 2008 | Hong | Korea | Asian | Schizophrenic patients | ? | M | N/A | homicide | group comparison, OAS, LHA | COMT-Val158Met | rs4680 | 193 |
| 2008 | Burt | USA | Caucasian | Students | 19.00 | M | N/A | Aggressive behavior | ASR | SLC6A3-40bpVNTR |  | 197 |
| 2008 | Burt | USA | Caucasian | Students | 19.00 | M | N/A | Aggressive behavior | ASR | TPH1-A218C | rs1800532 | 204 |
| 2008 | Ducci | USA | Indian-americans | Indian-american tribe (alcoholics +/- APD & controls) | 37.80 | F | N/A | APD in alcoholism | group comparison (of ASPD) | MAOA promoter 30bpVNTR |  | 167 |
| 2008 | Gonzalez | Cuba | mixed | Students & staff |  | both | 24/76 | Aggression | KSP | CYP2D6 (various) | [various SNPs] | 246 |
| 2008 | Kim | Korea | Asian | S patients (& controls) | 38.40 | both | 96/69 | Aggressive behavior | group comparison, OAS | COMT-Val158Met | rs4680 | 167 |
| 2008 | Pombo | Portugal | Caucasian | Alcohol dependent | 48.00 | both | 78/19 | outward aggression | SDAS | 5HTTLPR | rs4795541 | 97 |
| 2009 | Gonda | Hungary | Caucasian | Healthy volunteers | 32.13 | F | N/A | aggression, hostility | BDHI | 5HTTLPR | rs4795541 | 169 |
| 2009 | Tikkanen | Finland | Caucasian | alcoholic criminals | 32.50 | M | N/A | Impulsive violence | examination | MAOA promoter 30bpVNTR |  | 174 |
| 2009 | Sysoeva | Russia | Caucasian | Young women, controls | 13.00 | F | N/A | Aggressive behavior | BDHI | 5HTTLPR | rs4795541 | 64 |
| 2009 | Sysoeva | Russia | Caucasian | Young women, controls | 23.00 | F | N/A | Aggressive behavior | BDHI | 5HTTLPR | rs4795541 | 40 |
| 2009 | Sysoeva | Russia | Caucasian | Young women, swimmers | 13.00 | F | N/A | Aggressive behavior | BDHI | 5HTTLPR | rs4795541 | 62 |
| 2009 | Reif | Germany | Caucasian | forensic referrals (violent/no violent) | 34.10 | M | N/A | Violence | group comparison | NOS1 Ex1f VNTR |  | 182 |
| 2009 | McDermott | USA | mixed | Students | 22.00 | M | N/A | Aggressive tendencies | controlled experiment | MAOA promoter 30bpVNTR |  | 70 |
| 2009 | Williams | Australia | Caucasian | Healthy individuals | 36.34 | M | N/A | Antisocial index | NEO | MAOA promoter 30bpVNTR |  | 140 |
| 2009 | Williams | Australia | Caucasian | Healthy individuals | 35.80 | F | N/A | Antisocial index | NEO | MAOA promoter 30bpVNTR |  | 68 |
| 2009 | Terraciano | Italy | Caucasian | volunteers | 42.60 | both | 43/57 | anger-hostility | NEO | 5HTTLPR | rs4795541 | 3913 |
| 2009 | Terraciano | USA | mixed | volunteers | 52.90 | both | 49/51 | anger-hostility | NEO | 5HTTLPR | rs4795541 | 548 |
| 2009 | Conner | USA | Caucasian | Students | 18.70 | both | 168/193 | Hostility questionnaire | controlled experiment | HTR1B-G861C | rs6296 | 361 |
| 2009 | Kinnally | USA | mixed | mood disorders & controls | 38.60 | F | N/A | Hostility aggressiveness | BGAI, BIS, BDHI | MAOA promoter 30bpVNTR |  | 159 |
| 2009 | Kim | Korea | Asian | Schizophrenic patients | 38.20 | both | 56/47 | aggression, anger | MOAS, STAXI, group comparison | 5HTTLPR | rs4795541 | 103 |
| 2009 | Kim | Korea | Asian | Schizophrenic patients | 38.20 | both |  | aggression, anger | MOAS, STAXI, group comparison | 5HTTLPR | rs4795541 |  |
| 2009 | Gu | China | Asian | Schizophrenic patients | 36.00 | M | N/A | Violent behavior | MOAS, group comparison | COMT-Val158Met | rs4680 | 584 |
| 2009 | Wagner | Germany | Caucasian | Borderline PD | 33.00 | both | 49/110 | impulsive aggression | BDHI | BDNF-Val66Met | rs6265 | 159 |
| 2009 | Wagner | Germany | Caucasian | Borderline PD | 32.00 | F | N/A | impulsive aggression | BDHI | COMT-Val158Met | rs4680 | 161 |
| 2009 | Edwards | USA | Caucasian | School children from general population |  | M | N/A | externalizing behavior | Child behavior checklist | MAOA promoter 30bpVNTR |  | 186 |
| 2009 | Perroud | France | ? | Suicide attempters | 39.60 | both | 30/70 | Anger | STAXI | COMT-Val158Met | rs4680 | 779 |
| 2009 | Perroud | France | ? | Suicide attempters | 39.60 | both | 30/70 | Anger | STAXI | 5HTTLPR | rs4795541 | 835 |
| 2009 | Perroud | France | ? | Suicide attempters | 39.60 | both | 30/70 | Anger | STAXI | TPH1-A218C | rs1800532 | 840 |
| 2009 | Perroud | France | ? | Suicide attempters | 39.60 | both | 30/70 | Anger | STAXI | HTR2A-1438A/G | rs6311 | 562 |
| 2009 | Perroud | France | ? | Suicide attempters | 39.60 | M | N/A | Anger | STAXI | MAOA promoter 30bpVNTR |  | 248 |
| 2009 | Perroud | France | ? | Suicide attempters | 39.60 | F | N/A | Anger | STAXI | MAOA promoter 30bpVNTR |  | 590 |
| 2009 | Perroud | France | ? | Suicide attempters | 39.60 | both | 30/70 | Anger | STAXI | BDNF-Val66Met | rs6265 | 842 |
| 2009 | Perroud | France | ? | Suicide attempters | 39.60 | both | 30/70 | Anger | STAXI | HTR1B-G861C | rs6296 | 839 |
| 2009 | Kim | Korea | Asian | Schizophrenic patients | 38.20 | both | 57/47 | aggression, anger | group comparison, OAS, STAXI | TPH1-A218C | rs1800532 | 104 |
| 2009 | Hohmann | Germany | Caucasian | Adolescents | 15.00 | both | 144/154 | externalizing behavior | YSR, CBCL | 5HTTLPR | rs4795541 | 298 |
| 2009 | Hohmann | Germany | Caucasian | Adolescents | 15.00 | both | 144/154 | externalizing behavior | YSR, CBCL | DRD4-ex3 48bp VNTR |  | 298 |
| 2009 | Zimmerman | Germany | Caucasian | Adolescents | 12.00 | both | 45/46 | aggressiveness | CCQ (mothers report) | 5HTTLPR | rs4795541 | 91 |
| 2009 | May | USA | Caucasian | Intellectual disabilities (& controls) | 41.00 | M | N/A | Problem behavior | group comparison, N of PB | MAOA promoter 30bpVNTR |  | 70 |
| 2009 | Jensen | USA | Caucasian | Students | 18.60 | both |  | Conduct disorder items | Conduct disorder items | HTR1B | rs13212041 | 359 |
| 2009 | Westberg | Sweden | Caucasian | volunteers | 59.00 | M | N/A | nonconformity factor (KSP) | KSP, TCI | AR_(CAG)n | rs72181988 | 141 |
| 2009 | Westberg | Sweden | Caucasian | forensic patients with violent history | 35.00 | M | N/A | nonconformity factor (KSP) | KSP, TCI | AR_(CAG)n | rs72181988 | 63 |
| 2010 | Isir | Turkey | Caucasian | Violent offenders with MR vs controls | 13.50 | both | 45/27 | violent offence | group comparison | COMT-Val158Met | rs4680 | 72 |
| 2010 | Kim | Korea | Asian | S patients (& controls) | 38.40 | both | 96/69 | aggression, anger | group comparison, OAS, STAXI | TPH1-A218C | rs1800532 | 165 |
| 2010 | Beaver | USA | mixed | adolescents | 16.47 | M | N/A | gang membership, weapon use | group comparison | MAOA promoter 30bpVNTR |  | 1041 |
| 2010 | Beaver | USA | mixed | adolescents | 16.47 | F | N/A | gang membership, weapon use | group comparison | MAOA promoter 30bpVNTR |  | 1155 |
| 2010 | Dmitrieva | Russia | Caucasian | adolescents | 14.60 | both | 50/50 | Delinquency | Problem behavior scale & offending checklist | DRD4-ex3 48bp VNTR |  | 235 |
| 2010 | Perez-Rodriguez | USA | Caucasian | PD and controls | 42.00 | both | 70/53 | Aggression | BDHI, BPAQ, OAS | TPH2 |  | 123 |
| 2010 | Chung | Korea | Asian | violent vs non-violent S patients | 38.00 | M | N/A | homicide | group comparison, LHA, OAS | BDNF-Val66Met | rs6265 | 95 |
| 2010 | Williams | USA | mixed | Healthy volunteers | 34.20 | M | N/A | Hostility | Cook-Medley, Buss-Perry, HDHQ | MAOA promoter 30bpVNTR |  | 86 |
| 2010 | Yang | Korea | Asian | Students | 23.00 | F | N/A | Anger | STAXI | 5HTTLPR | rs4795541 | 228 |
| 2010 | Albaugh | USA | ? | Children | 11.00 | both | 97/69 | Aggressive behavior | Child behavior checklist | COMT-Val158Met | rs4680 | 149 |
| 2010 | May | USA | Caucasian | Intellectual disabilities (& controls) | 41.00 | M | N/A | Problem behavior | group comparison, N of PB | 5HTTLPR | rs4795541 | 67 |
| 2010 | May | USA | Caucasian | Intellectual disabilities (& controls) | 41.00 | M | N/A | Problem behavior | group comparison, N of PB | 5HTT-VNTR |  | 63 |
| 2010 | Grigorenko | Russia | Caucasian | Adolescents | 16.20 | M | N/A | Incancerated offenders vs controls | group comparison | COMT-Val158Met | rs4680 | 461 |
| 2011 | Koh | Korea | Asian | Violent S & controls | 34.00 | both | 138/94 | criminal activity | group comparison, LHA | COMT-Val158Met | rs4680 | 229 |
| 2011 | Koh | Korea | Asian | Violent S & controls | 34.00 | both | 138/94 | criminal activity | group comparison, LHA | TPH1-A218C | rs1800532 | 230 |
| 2011 | Kasiakogia | UK | ? | Alcoholics with criminal record (& controls) |  |  |  | criminal police record | group comparison | DRD2-Taq1A | rs1800497 | 1006 |
| 2011 | Vogel | Germany | Caucasian | BPD patients & controls | 33.20 | both | 95/223 | impulsive aggression | group comparison, BDHI | AVPR1A |  | 318 |
| 2011 | Johansson | Finland | Caucasian | University students administered alcohol or placebo | 22.70 | M | N/A | Aggressive behavior | laboratory paradigm | OXTR |  | 116 |
| 2011 | Fergusson | New Zealand | mixed | birth cohort - not exposed to abuse | 30.00 | M | N/A | Violent offences | self-report, police, SCL-90 | MAOA promoter 30bpVNTR |  | 309 |
| 2011 | Fergusson | New Zealand | mixed | birth cohort - exposed to abuse | 30.00 | M | N/A | Violent offences | self-report, police, SCL-90 | MAOA promoter 30bpVNTR |  | 89 |
| 2011 | Gonda | Hungary | Caucasian | depressed | 48.00 | F | N/A | Aggression | BDHI | 5HTTLPR | rs4795541 | 137 |
| 2011 | Gonda | Hungary | Caucasian | controls | 32.00 | F | N/A | Aggression | BDHI | 5HTTLPR | rs4795541 | 118 |
| 2011 | Tosato | Italy | Caucasian | S patients | 42.10 | both | 41/39 | Aggression against others | OAS, group comparison | COMT-Val158Met | rs4680 | 80 |
| 2011 | Aluja | Spain | Caucasian | Inmates (& vs controls) |  | M | N/A | aggression hostility | Zuckerman questionnaire | AR_(CAG)n | rs72181988 | 153 |
| 2011 | Aluja | Spain | Caucasian | controls |  | M | N/A | aggression hostility | Zuckerman questionnaire | AR_(CAG)n | rs72181988 | 108 |
| 2011 | Brennan | Australia | Caucasian | birth cohort | 20.00 | both | 43/57 | Aggression | ABCL, ASR | COMT-Val158Met | rs4680 | 430 |
| 2011 | Reti | USA | mixed | cohort |  | both | 224/211 | antisocial behavior | ASPD, NEO | MAOA promoter 30bpVNTR |  | 435 |
| 2011 | Zai | Canada | mixed | children with pervasive aggression vs controls | 10.80 | both | 104/40 | >90 percentile of aggression | CBC & TRF | SLC6A3-40bpVNTR |  | 260 |
| 2011 | Zai | Canada | mixed | children with pervasive aggression vs controls | 10.80 | both | 104/40 | >90 percentile of aggression | CBC & TRF | DRD2-Taq1A | rs1800497 | 282 |
| 2011 | Zai | Canada | mixed | children with pervasive aggression vs controls | 10.80 | both | 104/40 | >90 percentile of aggression | CBC & TRF | DRD4-ex3 48bp VNTR |  | 256 |
| 2011 | Hurd | Canada | mixed | psychology students | 19.20 | M | N/A | physical aggression | AQ | AR_(CAG)n | rs72181988 |  |
| 2011 | Hurd | Canada | mixed | psychology students | 19.20 | M | N/A | physical aggression | AQ | MAOA promoter 30bpVNTR |  |  |
| 2011 | Zalsman | Israel | Caucasian | suicidal and not suicidal adolescents | 16.00 | both |  | Anger, Impulse control | ICS, Anger inventory | HTR2A, 5HTTLPR, MAO-A |  | 211 |
| 2011 | Nedic | Croatia | Caucasian | suicidal alcoholics | 49.50 | M | N/A | Aggressive behavior | BGLHA | COMT-Val158Met | rs4680 | 59 |
| 2011 | Nedic | Croatia | Caucasian | not suicidal alcoholics | 49.50 | M | N/A | Aggressive behavior | BGLHA | COMT-Val158Met | rs4680 | 253 |
| 2011 | Nedic | Croatia | Caucasian | suicidal alcoholics | 51.50 | F | N/A | Aggressive behavior | BGLHA | COMT-Val158Met | rs4680 | 23 |
| 2011 | Nedic | Croatia | Caucasian | not suicidal alcoholics | 51.50 | F | N/A | Aggressive behavior | BGLHA | COMT-Val158Met | rs4680 | 58 |
| 2011 | Basoglu | Turkey | Caucasian | offenders with APD & controls | 21.00 | M | N/A | offending behavior | group comparison, Psychopathy | SNAP25 DdeI |  | 129 |
| 2011 | Cao | China | Asian | Children exposed to domestic violence | 14.50 | both |  | Aggressive behavior | group comparison, CBC | COMT-Val158Met | rs4680 | 68 |
| 2011 | Cao | China | Asian | Children exposed to domestic violence | 14.50 | both |  | Aggressive behavior | group comparison, CBC | 5HTTLPR | rs4795541 | 68 |

**Supplementary Table 2.** Grading of the strength of cumulative evidence of meta-analyses with nominally positive findings according to the HuGENet criteria.

| **Gene** | **Subgroup*** | **Model** | **N** | **AMOUNT** | **N minor** | **REPLICATION** | **I^2^** | **BIAS** | **Publ. Bias** | **OVERALL** |
| --- | --- | --- | --- | --- | --- | --- | --- | --- | --- | --- |
| Studies with categorical outcomes | | |  |  |  |  |  |  |  |  |
| COMT | Violence | allelic | 6 | B | 344 | C | 54 | C | Harbord p=0.047 | C |
| MAOA-M | Violence | allelic | 3 | B | 629 | A | 0 | A |  | B |
| TPH1 | Psychiatric patients | allelic | 4 | B | 772 | A | 16 | B |  | B |
| Studies with continuous outcomes | | |  |  |  |  |  |  |  |  |
| 5HTTLPR | Age >16 and <65 | dominant | 22 | A | 9742 | B | 39 | C | Egger p = 0.044 | C |
| 5HTTLPR | Substance users | dominant | 4 | B | 599 | B | 28 | C | Egger p = 0.027 | C |
| COMT | Caucasian | recessive | 16 | A | 4933 | C | 56 | B |  | C |
| COMT | Substance users | recessive | 4 | B | 378 | A | 12 | B |  | B |
| COMT | Anger | recessive | 5 | A | 1355 | A | 0 | B |  | B |
| COMT | Caucasian | additive | 16 | A | 4933 | C | 54 | B |  | C |
| COMT | Substance users | additive | 4 | B | 378 | A | 0 | B |  | B |
| MAOA-F | Aggressiveness | recessive | 4 | B | 263 | A | 0 | B |  | B |

* Subgroup analyses were performed by ethnicity, age of participants, sample characteristics, and outcome measure.

N is the number of studies included, N minor, the size of the smallest genetic group (minor allele), I^2^ = I square test for heterogeneity and Publ. bias = the method and the p-value of publication bias (when significant). The overall evidence was rated as A (strong), B (moderate) or C (weak).

**Supplementary Figure 1.** Forest Plots of meta-analyses of studies with categorical outcomes

**5HT-1B-G861C**

1. By ethnicity (1 caucasian, 0 other)

4. By outcome measure

**5HTTLPR**

1. By ethnicity (1: caucasian, 0: other)

2. By Age (1: <16, 2: 16-65, 3: >65)

3. By sample characteristics

4. By outcome measure

**5HTT-VNTR**

**BDNF (val/met)**

2. By Age (1: <16, 2: 16-65, 3: >65)

**COMT**

1. By ethnicity (1: caucasian, 0: other)

2. By Age (1: <16, 2: 16-65, 3: >65)

3. By sample characteristics

4. By outcome measure

**DAT1_gen**

**DRD4_gen**

2. By Age (1: <16, 2: 16-65, 3: >65)

**MAO-F**

1. By ethnicity (1: caucasian, 0: other)

3. By sample characteristics

4. By outcome measure

**MAO-M**

1. By ethnicity (1: caucasian, 0: other)

2. By Age (1: <16, 2: 16-65, 3: >65)

3. By sample characteristics

4. By outcome measure

**TPH**

1. By ethnicity (1: caucasian, 0: other)

2. By Age (1: <16, 2: 16-65, 3: >65)

3. By sample characteristics

**AR (CAG)**

**DRD2**

**Supplementary Figure 2.** Forest Plots of meta-analyses of studies with continuous outcomes

**5HT-2A A1438G** Dominant

**5HT-2A A1438G** Recessive

**5HT-2A A1438G** Additive

**5HTTLPR** Dominant

1. By ethnicity (1: caucasian, 0: other)

2. By Age (1: <16, 2: 16-65, 3: >65)

3. By sample characteristics

4. By outcome measure

**5HTTLPR** Recessive

1. By ethnicity (1: caucasian, 0: other)

2. By Age (1: <16, 2: 16-65, 3: >65)

3. By sample characteristics

4. By outcome measure

**5HTTLPR** Additive

1. By ethnicity (1: caucasian, 0: other)

2. By Age (1: <16, 2: 16-65, 3: >65)

3. By sample characteristics

4. By outcome measure

**BDNF**

**COMT** Dominant

1. By ethnicity (1: caucasian, 0: other)

2. By Age (1: <16, 2: 16-65, 3: >65)

3. By sample characteristics

4. By outcome measure

**COMT** Recessive

1. By ethnicity (1: caucasian, 0: other)

2. By Age (1: <16, 2: 16-65, 3: >65)

3. By sample characteristics

4. By outcome measure

**COMT** Additive

1. By ethnicity (1: caucasian, 0: other)

2. By Age (1: <16, 2: 16-65, 3: >65)

3. By sample characteristics

4. By outcome measure

**DAT1** Dominant

2. By Age (1: <16, 2: 16-65, 3: >65)

3. By sample characteristics

**DAT1** Recessive

**DAT1** Additive

**DRD4-ex3** Dominant

1. By ethnicity (1: caucasian, 0: other)

2. By Age (1: <16, 2: 16-65, 3: >65)

3. By sample characteristics

4. By outcome measure

**MAO-Females** Dominant

1. By ethnicity (1: caucasian, 0: other)

2. By Age (1: <16, 2: 16-65, 3: >65)

3. By sample characteristics

4. By outcome measure

**MAO-Females** Recessive

1. By ethnicity (1: caucasian, 0: other)

3. By sample characteristics

4. By outcome measure

**MAO-Females** Additive

1. By ethnicity (1: caucasian, 0: other)

3. By sample characteristics

4. By outcome measure

**MAO-Males**

1. By ethnicity (1: caucasian, 0: other)

2. By Age (1: <16, 2: 16-65, 3: >65)

3. By sample characteristics

4. By outcome measure

**TPH** Dominant

1. By ethnicity (1: caucasian, 0: other)

3. By sample characteristics

4. By outcome measure

**TPH** Recessive

1. By ethnicity (1: caucasian, 0: other)

3. By sample characteristics

4. By outcome measure

**TPH** Additive

1. By ethnicity (1: caucasian, 0: other)

3. By sample characteristics

4. By outcome measure

**AR (CAG)**

**References**

1. Buss AH, Durkee A. An inventory for assessing different kinds of hostility. *J Consult Psychol* 1957; **21**(4)**:** 343-349.

2. Yudofsky SC, Silver JM, Jackson W, Endicott J, Williams D. The Overt Aggression Scale for the objective rating of verbal and physical aggression. *Am J Psychiatry* 1986; **143**(1)**:** 35-39.

3. Brown GL, Goodwin FK, Ballenger JC, Goyer PF, Major LF. Aggression in humans correlates with cerebrospinal fluid amine metabolites. *Psychiatry Res* 1979; **1**(2)**:** 131-139.

4. Reisberg B, Borenstein J, Salob SP, Ferris SH, Franssen E, Georgotas A. Behavioral symptoms in Alzheimer's disease: phenomenology and treatment. *J Clin Psychiatry* 1987; **48 Suppl:** 9-15.

5. Plutchik R, van Praag HM. A self-report measure of violence risk, II. *Compr Psychiatry* 1990; **31**(5)**:** 450-456.

6. Cummings JL, Mega M, Gray K, Rosenberg-Thompson S, Carusi DA, Gornbein J. The Neuropsychiatric Inventory: comprehensive assessment of psychopathology in dementia. *Neurology* 1994; **44**(12)**:** 2308-2314.

7. Achenbach TM. *Manual for the child behavior checklist/4-18 and 1991 profile*. Dept. of Psychiatry, University of Vermont: Burlington, Vt., 1991, x, 288 p.pp.

8. Quay HC, & Peterson, D. R. *The Revised Behavior Problem Checklist: Manual*. Psychological Assessment Resources: Odessa, FL, 1996.

9. Buss AH, Perry M. The aggression questionnaire. *J Pers Soc Psychol* 1992; **63**(3)**:** 452-459.

10. Coccaro EF, Berman ME, Kavoussi RJ. Assessment of life history of aggression: development and psychometric characteristics. *Psychiatry Res* 1997; **73**(3)**:** 147-157.

11. Spielberger C. *Manual for the State-Trait Anger Expression Inventory (STAXI)*. Psychological Assessment Resources: Odessa, FL, 1988.

12. Costa PT Jr. M, RR. *Revised NEO Personality Inventory (NEO-PI-R) and NEO Five-Factor Inventory (NEO-FFI) professional manual.* Psychological Assessment Resources: Odessa, FL, 1992.

13. Allen NC, Bagade S, McQueen MB, Ioannidis JP, Kavvoura FK, Khoury MJ *et al.* Systematic meta-analyses and field synopsis of genetic association studies in schizophrenia: the SzGene database. *Nat Genet* 2008; **40**(7)**:** 827-834.

14. Purcell S, Cherny SS, Sham PC. Genetic Power Calculator: design of linkage and association genetic mapping studies of complex traits. *Bioinformatics* 2003; **19**(1)**:** 149-150.

15. DerSimonian R, Laird N. Meta-analysis in clinical trials. *Control Clin Trials* 1986; **7**(3)**:** 177-188.

16. Higgins JP, Thompson SG. Quantifying heterogeneity in a meta-analysis. *Stat Med* 2002; **21**(11)**:** 1539-1558.

17. Thorlund K, Imberger G, Johnston BC, Walsh M, Awad T, Thabane L *et al.* Evolution of heterogeneity (I2) estimates and their 95% confidence intervals in large meta-analyses. *PloS one* 2012; **7**(7)**:** e39471.

18. Higgins JP, Thompson SG, Deeks JJ, Altman DG. Measuring inconsistency in meta-analyses. *BMJ* 2003; **327**(7414)**:** 557-560.

19. Kavvoura FK, Ioannidis JP. Methods for meta-analysis in genetic association studies: a review of their potential and pitfalls. *Hum Genet* 2008; **123**(1)**:** 1-14.

20. Ioannidis JP. Interpretation of tests of heterogeneity and bias in meta-analysis. *J Eval Clin Pract* 2008; **14**(5)**:** 951-957.

21. Egger M, Davey Smith G, Schneider M, Minder C. Bias in meta-analysis detected by a simple, graphical test. *BMJ* 1997; **315**(7109)**:** 629-634.

22. Begg CB, Mazumdar M. Operating characteristics of a rank correlation test for publication bias. *Biometrics* 1994; **50**(4)**:** 1088-1101.

23. Harbord RM, Egger M, Sterne JA. A modified test for small-study effects in meta-analyses of controlled trials with binary endpoints. *Stat Med* 2006; **25**(20)**:** 3443-3457.

24. Ioannidis JP, Boffetta P, Little J, O'Brien TR, Uitterlinden AG, Vineis P *et al.* Assessment of cumulative evidence on genetic associations: interim guidelines. *Int J Epidemiol* 2008; **37**(1)**:** 120-132.

25. Albaugh MD, Harder VS, Althoff RR, Rettew DC, Ehli EA, Lengyel-Nelson T *et al.* COMT Val158Met genotype as a risk factor for problem behaviors in youth. *J Am Acad Child Adolesc Psychiatry* 2010; **49**(8)**:** 841-849.

26. Aluja A, Garcia LF, Blanch A, Fibla J. Association of androgen receptor gene, CAG and GGN repeat length polymorphism and impulsive-disinhibited personality traits in inmates: the role of short-long haplotype. *Psychiatric genetics* 2011; **21**(5)**:** 229-239.

27. Assal F, Alarcon M, Solomon EC, Masterman D, Geschwind DH, Cummings JL. Association of the serotonin transporter and receptor gene polymorphisms in neuropsychiatric symptoms in Alzheimer disease. *Archives of neurology* 2004; **61**(8)**:** 1249-1253.

28. Avramopoulos D, Stefanis NC, Hantoumi I, Smyrnis N, Evdokimidis I, Stefanis CN. Higher scores of self reported schizotypy in healthy young males carrying the COMT high activity allele. *Mol Psychiatry* 2002; **7**(7)**:** 706-711.

29. Baca-Garcia E, Vaquero C, Diaz-Sastre C, Garcia-Resa E, Saiz-Ruiz J, Fernandez-Piqueras J *et al.* Lack of association between the serotonin transporter promoter gene polymorphism and impulsivity or aggressive behavior among suicide attempters and healthy volunteers. *Psychiatry Res* 2004; **126**(2)**:** 99-106.

30. Basoglu C, Oner O, Ates A, Algul A, Bez Y, Cetin M *et al.* Synaptosomal-associated protein 25 gene polymorphisms and antisocial personality disorder: association with temperament and psychopathy. *Can J Psychiatry* 2011; **56**(6)**:** 341-347.

31. Beaver KM, DeLisi M, Vaughn MG, Barnes JC. Monoamine oxidase A genotype is associated with gang membership and weapon use. *Compr Psychiatry* 2010; **51**(2)**:** 130-134.

32. Beitchman JH, Baldassarra L, Mik H, De Luca V, King N, Bender D *et al.* Serotonin transporter polymorphisms and persistent, pervasive childhood aggression. *Am J Psychiatry* 2006; **163**(6)**:** 1103-1105.

33. Berggard C, Damberg M, Longato-Stadler E, Hallman J, Oreland L, Garpenstrand H. The serotonin 2A -1438 G/A receptor polymorphism in a group of Swedish male criminals. *Neurosci Lett* 2003; **347**(3)**:** 196-198.

34. Brennan PA, Hammen C, Sylvers P, Bor W, Najman J, Lind P *et al.* Interactions between the COMT Val108/158Met polymorphism and maternal prenatal smoking predict aggressive behavior outcomes. *Biological psychology* 2011; **87**(1)**:** 99-105.

35. Burt SA, Mikolajewski AJ. Preliminary evidence that specific candidate genes are associated with adolescent-onset antisocial behavior. *Aggress Behav* 2008; **34**(4)**:** 437-445.

36. Cadoret RJ, Langbehn D, Caspers K, Troughton EP, Yucuis R, Sandhu HK *et al.* Associations of the serotonin transporter promoter polymorphism with aggressivity, attention deficit, and conduct disorder in an adoptee population. *Compr Psychiatry* 2003; **44**(2)**:** 88-101.

37. Cao YP, Li LF, Zhao XF, Zhang YL. [Association between aggressive behaviors and COMT Val158Met and 5-HTTLPR polymorphisms in children]. *Zhongguo Dang Dai Er Ke Za Zhi* 2011; **13**(5)**:** 361-364.

38. Caspi A, Langley K, Milne B, Moffitt TE, O'Donovan M, Owen MJ *et al.* A replicated molecular genetic basis for subtyping antisocial behavior in children with attention-deficit/hyperactivity disorder. *Arch Gen Psychiatry* 2008; **65**(2)**:** 203-210.

39. Caspi A, McClay J, Moffitt TE, Mill J, Martin J, Craig IW *et al.* Role of genotype in the cycle of violence in maltreated children. *Science* 2002; **297**(5582)**:** 851-854.

40. Chen TJ, Blum K, Mathews D, Fisher L, Schnautz N, Braverman ER *et al.* Are dopaminergic genes involved in a predisposition to pathological aggression? Hypothesizing the importance of "super normal controls" in psychiatricgenetic research of complex behavioral disorders. *Med Hypotheses* 2005; **65**(4)**:** 703-707.

41. Cheng D, Hong CJ, Liao DL, Tsai SJ. Association study of androgen receptor CAG repeat polymorphism and male violent criminal activity. *Psychoneuroendocrinology* 2006; **31**(4)**:** 548-552.

42. Chung S, Chung HY, Jung J, Chang JK, Hong JP. Association among aggressiveness, neurocognitive function, and the Val66Met polymorphism of brain-derived neurotrophic factor gene in male schizophrenic patients. *Compr Psychiatry* 2010; **51**(4)**:** 367-372.

43. Comings DE, Johnson JP, Gonzalez NS, Huss M, Saucier G, McGue M *et al.* Association between the adrenergic alpha 2A receptor gene (ADRA2A) and measures of irritability, hostility, impulsivity and memory in normal subjects. *Psychiatr Genet* 2000; **10**(1)**:** 39-42.

44. Comings DE, Muhleman D, Ahn C, Gysin R, Flanagan SD. The dopamine D2 receptor gene: a genetic risk factor in substance abuse. *Drug Alcohol Depend* 1994; **34**(3)**:** 175-180.

45. Comings DE, Muhleman D, Johnson JP, MacMurray JP. Parent-daughter transmission of the androgen receptor gene as an explanation of the effect of father absence on age of menarche. *Child Dev* 2002; **73**(4)**:** 1046-1051.

46. Conner TS, Jensen KP, Tennen H, Furneaux HM, Kranzler HR, Covault J. Functional polymorphisms in the serotonin 1B receptor gene (HTR1B) predict self-reported anger and hostility among young men. *Am J Med Genet B Neuropsychiatr Genet* 2010; **153B**(1)**:** 67-78.

47. Craig D, Hart DJ, Carson R, McIlroy SP, Passmore AP. Allelic variation at the A218C tryptophan hydroxylase polymorphism influences agitation and aggression in Alzheimer's disease. *Neuroscience letters* 2004; **363**(3)**:** 199-202.

48. Damberg M, Garpenstrand H, Alfredsson J, Ekblom J, Forslund K, Rylander G *et al.* A polymorphic region in the human transcription factor AP-2beta gene is associated with specific personality traits. *Mol Psychiatry* 2000; **5**(2)**:** 220-224.

49. DeYoung CG, Peterson JB, Seguin JR, Mejia JM, Pihl RO, Beitchman JH *et al.* The dopamine D4 receptor gene and moderation of the association between externalizing behavior and IQ. *Arch Gen Psychiatry* 2006; **63**(12)**:** 1410-1416.

50. Dmitrieva J, Chen C, Greenberger E, Ogunseitan O, Ding YC. Gender-specific expression of the DRD4 gene on adolescent delinquency, anger and thrill seeking. *Soc Cogn Affect Neurosci* 2010; **6**(1)**:** 82-89.

51. Ducci F, Enoch MA, Hodgkinson C, Xu K, Catena M, Robin RW *et al.* Interaction between a functional MAOA locus and childhood sexual abuse predicts alcoholism and antisocial personality disorder in adult women. *Mol Psychiatry* 2008; **13**(3)**:** 334-347.

52. Edwards AC, Dodge KA, Latendresse SJ, Lansford JE, Bates JE, Pettit GS *et al.* MAOA-uVNTR and early physical discipline interact to influence delinquent behavior. *J Child Psychol Psychiatry* 2010; **51**(6)**:** 679-687.

53. Eisenberger NI, Way BM, Taylor SE, Welch WT, Lieberman MD. Understanding genetic risk for aggression: clues from the brain's response to social exclusion. *Biol Psychiatry* 2007; **61**(9)**:** 1100-1108.

54. Evans J, Reeves B, Platt H, Leibenau A, Goldman D, Jefferson K *et al.* Impulsiveness, serotonin genes and repetition of deliberate self-harm (DSH). *Psychol Med* 2000; **30**(6)**:** 1327-1334.

55. Fergusson DM, Boden JM, Horwood LJ, Miller AL, Kennedy MA. MAOA, abuse exposure and antisocial behaviour: 30-year longitudinal study. *Br J Psychiatry* 2011; **198**(6)**:** 457-463.

56. Flory JD, Xu K, New AS, Finch T, Goldman D, Siever LJ. Irritable assault and variation in the COMT gene. *Psychiatr Genet* 2007; **17**(6)**:** 344-346.

57. Foley DL, Eaves LJ, Wormley B, Silberg JL, Maes HH, Kuhn J *et al.* Childhood adversity, monoamine oxidase a genotype, and risk for conduct disorder. *Arch Gen Psychiatry* 2004; **61**(7)**:** 738-744.

58. Frazzetto G, Di Lorenzo G, Carola V, Proietti L, Sokolowska E, Siracusano A *et al.* Early trauma and increased risk for physical aggression during adulthood: the moderating role of MAOA genotype. *PLoS One* 2007; **2**(5)**:** e486.

59. Fresan A, Camarena B, Apiquian R, Aguilar A, Urraca N, Nicolini H. Association study of MAO-A and DRD4 genes in schizophrenic patients with aggressive behavior. *Neuropsychobiology* 2007; **55**(3-4)**:** 171-175.

60. Gelernter J, Kranzler H, Coccaro EF, Siever LJ, New AS. Serotonin transporter protein gene polymorphism and personality measures in African American and European American subjects. *Am J Psychiatry* 1998; **155**(10)**:** 1332-1338.

61. Gerra G, Garofano L, Bosari S, Pellegrini C, Zaimovic A, Moi G *et al.* Analysis of monoamine oxidase A (MAO-A) promoter polymorphism in male heroin-dependent subjects: behavioural and personality correlates. *J Neural Transm* 2004; **111**(5)**:** 611-621.

62. Gerra G, Garofano L, Castaldini L, Rovetto F, Zaimovic A, Moi G *et al.* Serotonin transporter promoter polymorphism genotype is associated with temperament, personality traits and illegal drugs use among adolescents. *J Neural Transm* 2005; **112**(10)**:** 1397-1410.

63. Gerra G, Garofano L, Pellegrini C, Bosari S, Zaimovic A, Moi G *et al.* Allelic association of a dopamine transporter gene polymorphism with antisocial behaviour in heroin-dependent patients. *Addict Biol* 2005; **10**(3)**:** 275-281.

64. Gerra G, Garofano L, Santoro G, Bosari S, Pellegrini C, Zaimovic A *et al.* Association between low-activity serotonin transporter genotype and heroin dependence: behavioral and personality correlates. *Am J Med Genet B Neuropsychiatr Genet* 2004; **126B**(1)**:** 37-42.

65. Gerra G, Zaimovic A, Garofano L, Ciusa F, Moi G, Avanzini P *et al.* Perceived parenting behavior in the childhood of cocaine users: relationship with genotype and personality traits. *Am J Med Genet B Neuropsychiatr Genet* 2007; **144B**(1)**:** 52-57.

66. Giegling I, Hartmann AM, Moller HJ, Rujescu D. Anger- and aggression-related traits are associated with polymorphisms in the 5-HT-2A gene. *J Affect Disord* 2006; **96**(1-2)**:** 75-81.

67. Giegling I, Rujescu D, Mandelli L, Schneider B, Hartmann AM, Schnabel A *et al.* Tachykinin receptor 1 variants associated with aggression in suicidal behavior. *Am J Med Genet B Neuropsychiatr Genet* 2007; **144B**(6)**:** 757-761.

68. Giegling I, Rujescu D, Mandelli L, Schneider B, Hartmann AM, Schnabel A *et al.* Estrogen receptor gene 1 variants are not associated with suicidal behavior. *Psychiatry Res* 2008; **160**(1)**:** 1-7.

69. Gietl A, Giegling I, Hartmann AM, Schneider B, Schnabel A, Maurer K *et al.* ABCG1 gene variants in suicidal behavior and aggression-related traits. *Eur Neuropsychopharmacol* 2007; **17**(6-7)**:** 410-416.

70. Goldman D, Dean M, Brown GL, Bolos AM, Tokola R, Virkkunen M *et al.* D2 dopamine receptor genotype and cerebrospinal fluid homovanillic acid, 5-hydroxyindoleacetic acid and 3-methoxy-4-hydroxyphenylglycol in alcoholics in Finland and the United States. *Acta Psychiatr Scand* 1992; **86**(5)**:** 351-357.

71. Gonda X, Fountoulakis KN, Csukly G, Bagdy G, Pap D, Molnar E *et al.* Interaction of 5-HTTLPR genotype and unipolar major depression in the emergence of aggressive/hostile traits. *Journal of affective disorders* 2011; **132**(3)**:** 432-437.

72. Gonda X, Fountoulakis KN, Juhasz G, Rihmer Z, Lazary J, Laszik A *et al.* Association of the s allele of the 5-HTTLPR with neuroticism-related traits and temperaments in a psychiatrically healthy population. *Eur Arch Psychiatry Clin Neurosci* 2009; **259**(2)**:** 106-113.

73. Gonzalez I, Penas-Lledo EM, Perez B, Dorado P, Alvarez M, A LL. Relation between CYP2D6 phenotype and genotype and personality in healthy volunteers. *Pharmacogenomics* 2008; **9**(7)**:** 833-840.

74. Greenberg BD, Li Q, Lucas FR, Hu S, Sirota LA, Benjamin J *et al.* Association between the serotonin transporter promoter polymorphism and personality traits in a primarily female population sample. *Am J Med Genet* 2000; **96**(2)**:** 202-216.

75. Grigorenko EL, De Young CG, Eastman M, Getchell M, Haeffel GJ, Klinteberg B *et al.* Aggressive behavior, related conduct problems, and variation in genes affecting dopamine turnover. *Aggress Behav* 2010; **36**(3)**:** 158-176.

76. Gu Y, Yun L, Tian Y, Hu Z. Association between COMT gene and Chinese male schizophrenic patients with violent behavior. *Med Sci Monit* 2009; **15**(9)**:** CR484-489.

77. Guerin AA, Beitchman JH, Strauss J, Kennedy JL. Association study of the brain-derived neurotrophic factor gene and childhood aggression. *Psychiatr Genet* 2007; **17**(1)**:** 7-8.

78. Guo G, Ou XM, Roettger M, Shih JC. The VNTR 2 repeat in MAOA and delinquent behavior in adolescence and young adulthood: associations and MAOA promoter activity. *Eur J Hum Genet* 2008; **16**(5)**:** 626-634.

79. Guo G, Roettger ME, Shih JC. Contributions of the DAT1 and DRD2 genes to serious and violent delinquency among adolescents and young adults. *Hum Genet* 2007; **121**(1)**:** 125-136.

80. Ha TM, Cho DM, Park SW, Joo MJ, Lee BJ, Kong BG *et al.* Evaluating associations between 5-HTTLPR polymorphism and Alzheimer's disease for Korean patients. *Dement Geriatr Cogn Disord* 2005; **20**(1)**:** 31-34.

81. Haberstick BC, Lessem JM, Hopfer CJ, Smolen A, Ehringer MA, Timberlake D *et al.* Monoamine oxidase A (MAOA) and antisocial behaviors in the presence of childhood and adolescent maltreatment. *Am J Med Genet B Neuropsychiatr Genet* 2005; **135B**(1)**:** 59-64.

82. Haberstick BC, Smolen A, Hewitt JK. Family-based association test of the 5HTTLPR and aggressive behavior in a general population sample of children. *Biol Psychiatry* 2006; **59**(9)**:** 836-843.

83. Hallikainen T, Lachman H, Saito T, Volavka J, Kauhanen J, Salonen JT *et al.* Lack of association between the functional variant of the catechol-o-methyltransferase (COMT) gene and early-onset alcoholism associated with severe antisocial behavior. *Am J Med Genet* 2000; **96**(3)**:** 348-352.

84. Hallikainen T, Saito T, Lachman HM, Volavka J, Pohjalainen T, Ryynanen OP *et al.* Association between low activity serotonin transporter promoter genotype and early onset alcoholism with habitual impulsive violent behavior. *Mol Psychiatry* 1999; **4**(4)**:** 385-388.

85. Han DH, Kee BS, Min KJ, Lee YS, Na C, Park DB *et al.* Effects of catechol-O-methyltransferase Val158Met polymorphism on the cognitive stability and aggression in the first-onset schizophrenic patients. *Neuroreport* 2006; **17**(1)**:** 95-99.

86. Han DH, Park DB, Na C, Kee BS, Lee YS. Association of aggressive behavior in Korean male schizophrenic patients with polymorphisms in the serotonin transporter promoter and catecholamine-O-methyltransferase genes. *Psychiatry Res* 2004; **129**(1)**:** 29-37.

87. Hill EM, Stoltenberg SF, Bullard KH, Li S, Zucker RA, Burmeister M. Antisocial alcoholism and serotonin-related polymorphisms: association tests. *Psychiatr Genet* 2002; **12**(3)**:** 143-153.

88. Hohmann S, Becker K, Fellinger J, Banaschewski T, Schmidt MH, Esser G *et al.* Evidence for epistasis between the 5-HTTLPR and the dopamine D4 receptor polymorphisms in externalizing behavior among 15-year-olds. *J Neural Transm* 2009; **116**(12)**:** 1621-1629.

89. Holmes C, Smith H, Ganderton R, Arranz M, Collier D, Powell J *et al.* Psychosis and aggression in Alzheimer's disease: the effect of dopamine receptor gene variation. *J Neurol Neurosurg Psychiatry* 2001; **71**(6)**:** 777-779.

90. Hong JP, Lee JS, Chung S, Jung J, Yoo HK, Chang SM *et al.* New functional single nucleotide polymorphism (Ala72Ser) in the COMT gene is associated with aggressive behavior in male schizophrenia. *Am J Med Genet B Neuropsychiatr Genet* 2008; **147B**(5)**:** 658-660.

91. Huang YY, Cate SP, Battistuzzi C, Oquendo MA, Brent D, Mann JJ. An association between a functional polymorphism in the monoamine oxidase a gene promoter, impulsive traits and early abuse experiences. *Neuropsychopharmacology* 2004; **29**(8)**:** 1498-1505.

92. Huang YY, Grailhe R, Arango V, Hen R, Mann JJ. Relationship of psychopathology to the human serotonin1B genotype and receptor binding kinetics in postmortem brain tissue. *Neuropsychopharmacology* 1999; **21**(2)**:** 238-246.

93. Huizinga D, Haberstick BC, Smolen A, Menard S, Young SE, Corley RP *et al.* Childhood maltreatment, subsequent antisocial behavior, and the role of monoamine oxidase A genotype. *Biol Psychiatry* 2006; **60**(7)**:** 677-683.

94. Hurd PL, Vaillancourt KL, Dinsdale NL. Aggression, digit ratio and variation in androgen receptor and monoamine oxidase a genes in men. *Behav Genet* 2011; **41**(4)**:** 543-556.

95. Ishiguro H, Saito T, Akazawa S, Mitushio H, Tada K, Enomoto M *et al.* Association between drinking-related antisocial behavior and a polymorphism in the serotonin transporter gene in a Japanese population. *Alcohol Clin Exp Res* 1999; **23**(7)**:** 1281-1284.

96. Isir AB, Dai AI, Nacak M, Gorucu S. Study: the lack of significant association of the catechol-O-methyl transferase (COMT) gene polymorphism in violent offenders with mental retardation. *J Forensic Sci* 2010; **55**(1)**:** 225-228.

97. Jacob CP, Muller J, Schmidt M, Hohenberger K, Gutknecht L, Reif A *et al.* Cluster B personality disorders are associated with allelic variation of monoamine oxidase A activity. *Neuropsychopharmacology* 2005; **30**(9)**:** 1711-1718.

98. Jensen KP, Covault J, Conner TS, Tennen H, Kranzler HR, Furneaux HM. A common polymorphism in serotonin receptor 1B mRNA moderates regulation by miR-96 and associates with aggressive human behaviors. *Mol Psychiatry* 2009; **14**(4)**:** 381-389.

99. Johansson A, Bergman H, Corander J, Waldman ID, Karrani N, Salo B *et al.* Alcohol and aggressive behavior in men-moderating effects of oxytocin receptor gene (OXTR) polymorphisms. *Genes, brain, and behavior* 2012; **11**(2)**:** 214-221.

100. Jonsson EG, von Gertten C, Gustavsson JP, Yuan QP, Lindblad-Toh K, Forslund K *et al.* Androgen receptor trinucleotide repeat polymorphism and personality traits. *Psychiatr Genet* 2001; **11**(1)**:** 19-23.

101. Jorm AF, Prior M, Sanson A, Smart D, Zhang Y, Easteal S. Association of a polymorphism of the dopamine transporter gene with externalizing behavior problems and associated temperament traits: a longitudinal study from infancy to the mid-teens. *Am J Med Genet* 2001; **105**(4)**:** 346-350.

102. Kang JI, Namkoong K, Kim SJ. Association of DRD4 and COMT polymorphisms with anger and forgiveness traits in healthy volunteers. *Neurosci Lett* 2008; **430**(3)**:** 252-257.

103. Kasiakogia-Worlley K, McQuillin A, Lydall GJ, Patel S, Kottalgi G, Gunwardena P *et al.* Lack of allelic association between markers at the DRD2 and ANKK1 gene loci with the alcohol-dependence syndrome and criminal activity. *Psychiatric genetics* 2011; **21**(6)**:** 323-324.

104. Keltikangas-Jarvinen L, Puttonen S, Kivimaki M, Elovainio M, Pulkki-Raback L, Koivu M *et al.* Serotonin receptor genes 5HT1A and 5HT2A modify the relation between childhood temperament and adulthood hostility. *Genes Brain Behav* 2008; **7**(1)**:** 46-52.

105. Kim YR, Jahng JW, Min SK. Association between the serotonin transporter gene (5-HTTLPR) and anger-related traits in Korean schizophrenic patients. *Neuropsychobiology* 2009; **59**(3)**:** 165-171.

106. Kim YR, Kim JH, Kim SJ, Lee D, Min SK. Catechol-O-methyltransferase Val158Met polymorphism in relation to aggressive schizophrenia in a Korean population. *Eur Neuropsychopharmacol* 2008; **18**(11)**:** 820-825.

107. Kim YR, Lee JY, Min SK. No evidence of an association between A218C polymorphism of the tryptophan hydroxylase 1 gene and aggression in schizophrenia in a Korean population. *Yonsei Med J* 2010; **51**(1)**:** 27-32.

108. Kim-Cohen J, Caspi A, Taylor A, Williams B, Newcombe R, Craig IW *et al.* MAOA, maltreatment, and gene-environment interaction predicting children's mental health: new evidence and a meta-analysis. *Mol Psychiatry* 2006; **11**(10)**:** 903-913.

109. Kinnally EL, Huang YY, Haverly R, Burke AK, Galfalvy H, Brent DP *et al.* Parental care moderates the influence of MAOA-uVNTR genotype and childhood stressors on trait impulsivity and aggression in adult women. *Psychiatr Genet* 2009; **19**(3)**:** 126-133.

110. Koen L, Kinnear CJ, Corfield VA, Emsley RA, Jordaan E, Keyter N *et al.* Violence in male patients with schizophrenia: risk markers in a South African population. *Aust N Z J Psychiatry* 2004; **38**(4)**:** 254-259.

111. Koh KB, Choi EH, Lee YJ, Han M, Choi SS, Kim SW *et al.* The relation of serotonin-related gene and COMT gene polymorphisms with criminal behavior in schizophrenic disorder. *The Journal of clinical psychiatry* 2011.

112. Koller G, Bondy B, Preuss UW, Bottlender M, Soyka M. No association between a polymorphism in the promoter region of the MAOA gene with antisocial personality traits in alcoholics. *Alcohol Alcohol* 2003; **38**(1)**:** 31-34.

113. Kotler M, Barak P, Cohen H, Averbuch IE, Grinshpoon A, Gritsenko I *et al.* Homicidal behavior in schizophrenia associated with a genetic polymorphism determining low catechol O-methyltransferase (COMT) activity. *Am J Med Genet* 1999; **88**(6)**:** 628-633.

114. Kranzler HR, Hernandez-Avila CA, Gelernter J. Polymorphism of the 5-HT1B receptor gene (HTR1B): strong within-locus linkage disequilibrium without association to antisocial substance dependence. *Neuropsychopharmacology* 2002; **26**(1)**:** 115-122.

115. Kulikova MA, Maluchenko NV, Timofeeva MA, Shlepzova VA, Schegolkova JV, Sysoeva OV *et al.* Effect of functional catechol-O-methyltransferase Val158Met polymorphism on physical aggression. *Bull Exp Biol Med* 2008; **145**(1)**:** 62-64.

116. Kweon YS, Lee HK, Lee CT, Lee KU, Pae CU. Association of the serotonin transporter gene polymorphism with Korean male alcoholics. *J Psychiatr Res* 2005; **39**(4)**:** 371-376.

117. Kweon YS, Lee HK, Lee CT, Pae CU. Association study of catechol-O-methyltransferase gene polymorphism in Korean male alcoholics. *Psychiatr Genet* 2005; **15**(2)**:** 151-154.

118. Lachman HM, Nolan KA, Mohr P, Saito T, Volavka J. Association between catechol O-methyltransferase genotype and violence in schizophrenia and schizoaffective disorder. *Am J Psychiatry* 1998; **155**(6)**:** 835-837.

119. Lam LC, Tang NL, Ma SL, Zhang W, Chiu HF. 5-HT2A T102C receptor polymorphism and neuropsychiatric symptoms in Alzheimer's disease. *Int J Geriatr Psychiatry* 2004; **19**(6)**:** 523-526.

120. Lappalainen J, Long JC, Eggert M, Ozaki N, Robin RW, Brown GL *et al.* Linkage of antisocial alcoholism to the serotonin 5-HT1B receptor gene in 2 populations. *Arch Gen Psychiatry* 1998; **55**(11)**:** 989-994.

121. Lappalainen J, Long JC, Virkkunen M, Ozaki N, Goldman D, Linnoila M. HTR2C Cys23Ser polymorphism in relation to CSF monoamine metabolite concentrations and DSM-III-R psychiatric diagnoses. *Biol Psychiatry* 1999; **46**(6)**:** 821-826.

122. Liao DL, Hong CJ, Shih HL, Tsai SJ. Possible association between serotonin transporter promoter region polymorphism and extremely violent crime in Chinese males. *Neuropsychobiology* 2004; **50**(4)**:** 284-287.

123. Liou YJ, Tsai SJ, Hong CJ, Wang YC, Lai IC. Association analysis of a functional catechol-o-methyltransferase gene polymorphism in schizophrenic patients in Taiwan. *Neuropsychobiology* 2001; **43**(1)**:** 11-14.

124. Lu RB, Lin WW, Lee JF, Ko HC, Shih JC. Neither antisocial personality disorder nor antisocial alcoholism is associated with the MAO-A gene in Han Chinese males. *Alcohol Clin Exp Res* 2003; **27**(6)**:** 889-893.

125. Manuck SB, Flory JD, Ferrell RE, Dent KM, Mann JJ, Muldoon MF. Aggression and anger-related traits associated with a polymorphism of the tryptophan hydroxylase gene. *Biol Psychiatry* 1999; **45**(5)**:** 603-614.

126. Manuck SB, Flory JD, Ferrell RE, Mann JJ, Muldoon MF. A regulatory polymorphism of the monoamine oxidase-A gene may be associated with variability in aggression, impulsivity, and central nervous system serotonergic responsivity. *Psychiatry Res* 2000; **95**(1)**:** 9-23.

127. Manuck SB, Flory JD, Muldoon MF, Ferrell RE. Central nervous system serotonergic responsivity and aggressive disposition in men. *Physiol Behav* 2002; **77**(4-5)**:** 705-709.

128. Matsushita S, Kimura M, Miyakawa T, Yoshino A, Murayama M, Masaki T *et al.* Association study of brain-derived neurotrophic factor gene polymorphism and alcoholism. *Alcohol Clin Exp Res* 2004; **28**(11)**:** 1609-1612.

129. Matsushita S, Yoshino A, Murayama M, Kimura M, Muramatsu T, Higuchi S. Association study of serotonin transporter gene regulatory region polymorphism and alcoholism. *Am J Med Genet* 2001; **105**(5)**:** 446-450.

130. May ME, Lightfoot DA, Srour A, Kowalchuk RK, Kennedy CH. Association between serotonin transporter polymorphisms and problem behavior in adult males with intellectual disabilities. *Brain Res* 2010; **1357:** 97-103.

131. May ME, Srour A, Hedges LK, Lightfoot DA, Phillips JA, Blakely RD *et al.* Monoamine oxidase a promoter gene associated with problem behavior in adults with intellectual/developmental disabilities. *Am J Intellect Dev Disabil* 2009; **114**(4)**:** 269-273.

132. Mazzanti CM, Lappalainen J, Long JC, Bengel D, Naukkarinen H, Eggert M *et al.* Role of the serotonin transporter promoter polymorphism in anxiety-related traits. *Arch Gen Psychiatry* 1998; **55**(10)**:** 936-940.

133. McDermott R, Tingley D, Cowden J, Frazzetto G, Johnson DD. Monoamine oxidase A gene (MAOA) predicts behavioral aggression following provocation. *Proc Natl Acad Sci U S A* 2009; **106**(7)**:** 2118-2123.

134. Nedic G, Nikolac M, Sviglin KN, Muck-Seler D, Borovecki F, Pivac N. Association study of a functional catechol-O-methyltransferase (COMT) Val108/158Met polymorphism and suicide attempts in patients with alcohol dependence. *Int J Neuropsychopharmacol* 2011; **14**(3)**:** 377-388.

135. New AS, Gelernter J, Goodman M, Mitropoulou V, Koenigsberg H, Silverman J *et al.* Suicide, impulsive aggression, and HTR1B genotype. *Biol Psychiatry* 2001; **50**(1)**:** 62-65.

136. New AS, Gelernter J, Yovell Y, Trestman RL, Nielsen DA, Silverman J *et al.* Tryptophan hydroxylase genotype is associated with impulsive-aggression measures: a preliminary study. *Am J Med Genet* 1998; **81**(1)**:** 13-17.

137. Nielsen DA, Virkkunen M, Lappalainen J, Eggert M, Brown GL, Long JC *et al.* A tryptophan hydroxylase gene marker for suicidality and alcoholism. *Arch Gen Psychiatry* 1998; **55**(7)**:** 593-602.

138. Nilsson KW, Sjoberg RL, Damberg M, Leppert J, Ohrvik J, Alm PO *et al.* Role of monoamine oxidase A genotype and psychosocial factors in male adolescent criminal activity. *Biol Psychiatry* 2006; **59**(2)**:** 121-127.

139. Nobile M, Giorda R, Marino C, Carlet O, Pastore V, Vanzin L *et al.* Socioeconomic status mediates the genetic contribution of the dopamine receptor D4 and serotonin transporter linked promoter region repeat polymorphisms to externalization in preadolescence. *Dev Psychopathol* 2007; **19**(4)**:** 1147-1160.

140. Nolan KA, Volavka J, Lachman HM, Saito T. An association between a polymorphism of the tryptophan hydroxylase gene and aggression in schizophrenia and schizoaffective disorder. *Psychiatr Genet* 2000; **10**(3)**:** 109-115.

141. Parsian A, Cloninger CR. Serotonergic pathway genes and subtypes of alcoholism: association studies. *Psychiatr Genet* 2001; **11**(2)**:** 89-94.

142. Patkar AA, Berrettini WH, Hoehe M, Thornton CC, Gottheil E, Hill K *et al.* Serotonin transporter polymorphisms and measures of impulsivity, aggression, and sensation seeking among African-American cocaine-dependent individuals. *Psychiatry Res* 2002; **110**(2)**:** 103-115.

143. Perez-Rodriguez MM, Weinstein S, New AS, Bevilacqua L, Yuan Q, Zhou Z *et al.* Tryptophan-hydroxylase 2 haplotype association with borderline personality disorder and aggression in a sample of patients with personality disorders and healthy controls. *J Psychiatr Res* 2010; **44**(15)**:** 1075-1081.

144. Perlis RH, Purcell S, Fagerness J, Cusin C, Yamaki L, Fava M *et al.* Clinical and genetic dissection of anger expression and CREB1 polymorphisms in major depressive disorder. *Biol Psychiatry* 2007; **62**(5)**:** 536-540.

145. Perroud N, Jaussent I, Guillaume S, Bellivier F, Baud P, Jollant F *et al.* COMT but not serotonin-related genes modulates the influence of childhood abuse on anger traits. *Genes Brain Behav* 2010; **9**(2)**:** 193-202.

146. Persson ML, Wasserman D, Jonsson EG, Bergman H, Terenius L, Gyllander A *et al.* Search for the influence of the tyrosine hydroxylase (TCAT)(n) repeat polymorphism on personality traits. *Psychiatry Res* 2000; **95**(1)**:** 1-8.

147. Pombo S, de Quinhones Levy P, Bicho M, Barbosa A, Ismail F, Cardoso N. [Association of the functional serotonin transporter promoter polymorphism (5-HTTLPR) with externalizing and internalizing aggressivity and alcohol abuse]. *Acta Med Port* 2008; **21**(6)**:** 539-546.

148. Preuss UW, Koller G, Bondy B, Bahlmann M, Soyka M. Impulsive traits and 5-HT2A receptor promoter polymorphism in alcohol dependents: possible association but no influence of personality disorders. *Neuropsychobiology* 2001; **43**(3)**:** 186-191.

149. Pritchard AL, Harris J, Pritchard CW, Coates J, Haque S, Holder R *et al.* The effect of the apolipoprotein E gene polymorphisms and haplotypes on behavioural and psychological symptoms in probable Alzheimer's disease. *Journal of neurology, neurosurgery, and psychiatry* 2007; **78**(2)**:** 123-126.

150. Pritchard AL, Harris J, Pritchard CW, Coates J, Haque S, Holder R *et al.* Role of 5HT 2A and 5HT 2C polymorphisms in behavioural and psychological symptoms of Alzheimer's disease. *Neurobiol Aging* 2008; **29**(3)**:** 341-347.

151. Pritchard AL, Pritchard CW, Bentham P, Lendon CL. Role of serotonin transporter polymorphisms in the behavioural and psychological symptoms in probable Alzheimer disease patients. *Dementia and geriatric cognitive disorders* 2007; **24**(3)**:** 201-206.

152. Rajender S, Pandu G, Sharma JD, Gandhi KP, Singh L, Thangaraj K. Reduced CAG repeats length in androgen receptor gene is associated with violent criminal behavior. *Int J Legal Med* 2008; **122**(5)**:** 367-372.

153. Reif A, Jacob CP, Rujescu D, Herterich S, Lang S, Gutknecht L *et al.* Influence of functional variant of neuronal nitric oxide synthase on impulsive behaviors in humans. *Arch Gen Psychiatry* 2009; **66**(1)**:** 41-50.

154. Reif A, Rosler M, Freitag CM, Schneider M, Eujen A, Kissling C *et al.* Nature and nurture predispose to violent behavior: serotonergic genes and adverse childhood environment. *Neuropsychopharmacology* 2007; **32**(11)**:** 2375-2383.

155. Reist C, Mazzanti C, Vu R, Tran D, Goldman D. Serotonin transporter promoter polymorphism is associated with attenuated prolactin response to fenfluramine. *Am J Med Genet* 2001; **105**(4)**:** 363-368.

156. Reti IM, Xu JZ, Yanofski J, McKibben J, Uhart M, Cheng YJ *et al.* Monoamine oxidase A regulates antisocial personality in whites with no history of physical abuse. *Comprehensive psychiatry* 2011; **52**(2)**:** 188-194.

157. Retz W, Retz-Junginger P, Supprian T, Thome J, Rosler M. Association of serotonin transporter promoter gene polymorphism with violence: relation with personality disorders, impulsivity, and childhood ADHD psychopathology. *Behav Sci Law* 2004; **22**(3)**:** 415-425.

158. Retz W, Rosler M, Supprian T, Retz-Junginger P, Thome J. Dopamine D3 receptor gene polymorphism and violent behavior: relation to impulsiveness and ADHD-related psychopathology. *J Neural Transm* 2003; **110**(5)**:** 561-572.

159. Reuter M, Hennig J. Pleiotropic effect of the TPH A779C polymorphism on nicotine dependence and personality. *Am J Med Genet B Neuropsychiatr Genet* 2005; **134B**(1)**:** 20-24.

160. Rotondo A, Schuebel K, Bergen A, Aragon R, Virkkunen M, Linnoila M *et al.* Identification of four variants in the tryptophan hydroxylase promoter and association to behavior. *Mol Psychiatry* 1999; **4**(4)**:** 360-368.

161. Rujescu D, Giegling I, Bondy B, Gietl A, Zill P, Moller HJ. Association of anger-related traits with SNPs in the TPH gene. *Mol Psychiatry* 2002; **7**(9)**:** 1023-1029.

162. Rujescu D, Giegling I, Gietl A, Hartmann AM, Moller HJ. A functional single nucleotide polymorphism (V158M) in the COMT gene is associated with aggressive personality traits. *Biol Psychiatry* 2003; **54**(1)**:** 34-39.

163. Rujescu D, Giegling I, Mandelli L, Schneider B, Hartmann AM, Schnabel A *et al.* NOS-I and -III gene variants are differentially associated with facets of suicidal behavior and aggression-related traits. *Am J Med Genet B Neuropsychiatr Genet* 2008; **147B**(1)**:** 42-48.

164. Saito T, Lachman HM, Diaz L, Hallikainen T, Kauhanen J, Salonen JT *et al.* Analysis of monoamine oxidase A (MAOA) promoter polymorphism in Finnish male alcoholics. *Psychiatry Res* 2002; **109**(2)**:** 113-119.

165. Sakai JT, Lessem JM, Haberstick BC, Hopfer CJ, Smolen A, Ehringer MA *et al.* Case-control and within-family tests for association between 5HTTLPR and conduct problems in a longitudinal adolescent sample. *Psychiatr Genet* 2007; **17**(4)**:** 207-214.

166. Sakai JT, Young SE, Stallings MC, Timberlake D, Smolen A, Stetler GL *et al.* Case-control and within-family tests for an association between conduct disorder and 5HTTLPR. *Am J Med Genet B Neuropsychiatr Genet* 2006; **141B**(8)**:** 825-832.

167. Samochowiec J, Lesch KP, Rottmann M, Smolka M, Syagailo YV, Okladnova O *et al.* Association of a regulatory polymorphism in the promoter region of the monoamine oxidase A gene with antisocial alcoholism. *Psychiatry Res* 1999; **86**(1)**:** 67-72.

168. Sander T, Harms H, Dufeu P, Kuhn S, Hoehe M, Lesch KP *et al.* Serotonin transporter gene variants in alcohol-dependent subjects with dissocial personality disorder. *Biol Psychiatry* 1998; **43**(12)**:** 908-912.

169. Schulz-Heik RJ, Maentz SK, Rhee SH, Gelhorn HL, Young SE, Timberlake DS *et al.* Case-control and within-family tests for an association between conduct disorder and DAT1. *Psychiatr Genet* 2008; **18**(1)**:** 17-24.

170. Sen S, Nesse RM, Stoltenberg SF, Li S, Gleiberman L, Chakravarti A *et al.* A BDNF coding variant is associated with the NEO personality inventory domain neuroticism, a risk factor for depression. *Neuropsychopharmacology* 2003; **28**(2)**:** 397-401.

171. Sengupta SM, Grizenko N, Schmitz N, Schwartz G, Ben Amor L, Bellingham J *et al.* COMT Val108/158Met gene variant, birth weight, and conduct disorder in children with ADHD. *J Am Acad Child Adolesc Psychiatry* 2006; **45**(11)**:** 1363-1369.

172. Serretti A, Mandelli L, Giegling I, Schneider B, Hartmann AM, Schnabel A *et al.* HTR2C and HTR1A gene variants in German and Italian suicide attempters and completers. *Am J Med Genet B Neuropsychiatr Genet* 2007; **144B**(3)**:** 291-299.

173. Silva H, Iturra P, Solari A, Villarroel J, Jerez S, Vielma W *et al.* Serotonin transporter polymorphism and fluoxetine effect on impulsiveness and aggression in borderline personality disorder. *Actas Esp Psiquiatr* 2007; **35**(6)**:** 387-392.

174. Sjoberg RL, Nilsson KW, Wargelius HL, Leppert J, Lindstrom L, Oreland L. Adolescent girls and criminal activity: role of MAOA-LPR genotype and psychosocial factors. *Am J Med Genet B Neuropsychiatr Genet* 2007; **144B**(2)**:** 159-164.

175. Staner L, Uyanik G, Correa H, Tremeau F, Monreal J, Crocq MA *et al.* A dimensional impulsive-aggressive phenotype is associated with the A218C polymorphism of the tryptophan hydroxylase gene: a pilot study in well-characterized impulsive inpatients. *Am J Med Genet* 2002; **114**(5)**:** 553-557.

176. Stoltenberg SF, Twitchell GR, Hanna GL, Cook EH, Fitzgerald HE, Zucker RA *et al.* Serotonin transporter promoter polymorphism, peripheral indexes of serotonin function, and personality measures in families with alcoholism. *Am J Med Genet* 2002; **114**(2)**:** 230-234.

177. Strous RD, Bark N, Parsia SS, Volavka J, Lachman HM. Analysis of a functional catechol-O-methyltransferase gene polymorphism in schizophrenia: evidence for association with aggressive and antisocial behavior. *Psychiatry Res* 1997; **69**(2-3)**:** 71-77.

178. Strous RD, Nolan KA, Lapidus R, Diaz L, Saito T, Lachman HM. Aggressive behavior in schizophrenia is associated with the low enzyme activity COMT polymorphism: a replication study. *Am J Med Genet B Neuropsychiatr Genet* 2003; **120B**(1)**:** 29-34.

179. Sweet RA, Nimgaonkar VL, Kamboh MI, Lopez OL, Zhang F, DeKosky ST. Dopamine receptor genetic variation, psychosis, and aggression in Alzheimer disease. *Arch Neurol* 1998; **55**(10)**:** 1335-1340.

180. Sweet RA, Pollock BG, Sukonick DL, Mulsant BH, Rosen J, Klunk WE *et al.* The 5-HTTPR polymorphism confers liability to a combined phenotype of psychotic and aggressive behavior in Alzheimer disease. *Int Psychogeriatr* 2001; **13**(4)**:** 401-409.

181. Sysoeva OV, Maluchenko NV, Timofeeva MA, Portnova GV, Kulikova MA, Tonevitsky AG *et al.* Aggression and 5HTT polymorphism in females: study of synchronized swimming and control groups. *Int J Psychophysiol* 2009; **72**(2)**:** 173-178.

182. Terracciano A, Balaci L, Thayer J, Scally M, Kokinos S, Ferrucci L *et al.* Variants of the serotonin transporter gene and NEO-PI-R Neuroticism: No association in the BLSA and SardiNIA samples. *Am J Med Genet B Neuropsychiatr Genet* 2009; **150B**(8)**:** 1070-1077.

183. Tikkanen R, Sjoberg RL, Ducci F, Goldman D, Holi M, Tiihonen J *et al.* Effects of MAOA-genotype, alcohol consumption, and aging on violent behavior. *Alcohol Clin Exp Res* 2009; **33**(3)**:** 428-434.

184. Tosato S, Bonetto C, Di Forti M, Collier D, Cristofalo D, Bertani M *et al.* Effect of COMT genotype on aggressive behaviour in a community cohort of schizophrenic patients. *Neuroscience letters* 2011; **495**(1)**:** 17-21.

185. Tsai SJ, Liao DL, Yu YW, Chen TJ, Wu HC, Lin CH *et al.* A study of the association of (Val66Met) polymorphism in the brain-derived neurotrophic factor gene with alcohol dependence and extreme violence in Chinese males. *Neurosci Lett* 2005; **381**(3)**:** 340-343.

186. Twitchell GR, Hanna GL, Cook EH, Stoltenberg SF, Fitzgerald, He *et al.* Serotonin transporter promoter polymorphism genotype is associated with behavioral disinhibition and negative affect in children of alcoholics. *Alcohol Clin Exp Res* 2001; **25**(7)**:** 953-959.

187. Vanyukov MM, Moss HB, Yu LM, Deka R. A dinucleotide repeat polymorphism at the gene for monoamine oxidase A and measures of aggressiveness. *Psychiatry Res* 1995; **59**(1-2)**:** 35-41.

188. Verona E, Joiner TE, Johnson F, Bender TW. Gender specific gene-environment interactions on laboratory-assessed aggression. *Biol Psychol* 2006; **71**(1)**:** 33-41.

189. Vogel F, Wagner S, Baskaya O, Leuenberger B, Mobascher A, Dahmen N *et al.* Variable number of tandem repeat polymorphisms of the arginine vasopressin receptor 1A gene and impulsive aggression in patients with borderline personality disorder. *Psychiatric genetics* 2011.

190. Volavka J, Kennedy JL, Ni X, Czobor P, Nolan K, Sheitman B *et al.* COMT158 polymorphism and hostility. *Am J Med Genet B Neuropsychiatr Genet* 2004; **127B**(1)**:** 28-29.

191. Wagner S, Baskaya O, Anicker NJ, Dahmen N, Lieb K, Tadic A. The catechol o-methyltransferase (COMT) val(158)met polymorphism modulates the association of serious life events (SLE) and impulsive aggression in female patients with borderline personality disorder (BPD). *Acta Psychiatr Scand* 2010; **122**(2)**:** 110-117.

192. Wagner S, Baskaya O, Dahmen N, Lieb K, Tadic A. Modulatory role of the brain-derived neurotrophic factor Val66Met polymorphism on the effects of serious life events on impulsive aggression in borderline personality disorder. *Genes Brain Behav* 2010; **9**(1)**:** 97-102.

193. Wasserman D, Geijer T, Sokolowski M, Rozanov V, Wasserman J. Genetic variation in the hypothalamic-pituitary-adrenocortical axis regulatory factor, T-box 19, and the angry/hostility personality trait. *Genes Brain Behav* 2007; **6**(4)**:** 321-328.

194. Weder N, Yang BZ, Douglas-Palumberi H, Massey J, Krystal JH, Gelernter J *et al.* MAOA genotype, maltreatment, and aggressive behavior: the changing impact of genotype at varying levels of trauma. *Biol Psychiatry* 2009; **65**(5)**:** 417-424.

195. Wei J, Hemmings GP. Lack of evidence for association between the COMT locus and schizophrenia. *Psychiatr Genet* 1999; **9**(4)**:** 183-186.

196. Westberg L, Henningsson S, Landen M, Annerbrink K, Melke J, Nilsson S *et al.* Influence of androgen receptor repeat polymorphisms on personality traits in men. *J Psychiatry Neurosci* 2009; **34**(3)**:** 205-213.

197. Westberg L, Melke J, Landen M, Nilsson S, Baghaei F, Rosmond R *et al.* Association between a dinucleotide repeat polymorphism of the estrogen receptor alpha gene and personality traits in women. *Mol Psychiatry* 2003; **8**(1)**:** 118-122.

198. Widom CS, Brzustowicz LM. MAOA and the "cycle of violence:" childhood abuse and neglect, MAOA genotype, and risk for violent and antisocial behavior. *Biol Psychiatry* 2006; **60**(7)**:** 684-689.

199. Williams LM, Gatt JM, Kuan SA, Dobson-Stone C, Palmer DM, Paul RH *et al.* A polymorphism of the MAOA gene is associated with emotional brain markers and personality traits on an antisocial index. *Neuropsychopharmacology* 2009; **34**(7)**:** 1797-1809.

200. Williams RB, Surwit RS, Siegler IC, Ashley-Koch AE, Collins AL, Helms MJ *et al.* Central nervous system serotonin and clustering of hostility, psychosocial, metabolic, and cardiovascular endophenotypes in men. *Psychosom Med* 2010; **72**(7)**:** 601-607.

201. Yang J, Lee MS, Lee SH, Lee BC, Kim SH, Joe SH *et al.* Association between tryptophan hydroxylase 2 polymorphism and anger-related personality traits among young Korean women. *Neuropsychobiology* 2010; **62**(3)**:** 158-163.

202. Yang JW, Lee SH, Ryu SH, Lee BC, Kim SH, Joe SH *et al.* Association between monoamine oxidase A polymorphisms and anger-related personality traits in Korean women. *Neuropsychobiology* 2007; **56**(1)**:** 19-23.

203. Young SE, Smolen A, Corley RP, Krauter KS, DeFries JC, Crowley TJ *et al.* Dopamine transporter polymorphism associated with externalizing behavior problems in children. *Am J Med Genet* 2002; **114**(2)**:** 144-149.

204. Zai CC, Ehtesham S, Choi E, Nowrouzi B, de Luca V, Stankovich L *et al.* Dopaminergic system genes in childhood aggression: Possible role for DRD2. *World J Biol Psychiatry* 2012; **13**(1)**:** 65-74.

205. Zalsman G, Frisch A, Bromberg M, Gelernter J, Michaelovsky E, Campino A *et al.* Family-based association study of serotonin transporter promoter in suicidal adolescents: no association with suicidality but possible role in violence traits. *Am J Med Genet* 2001; **105**(3)**:** 239-245.

206. Zalsman G, Frisch A, King RA, Pauls DL, Grice DE, Gelernter J *et al.* Case control and family-based studies of tryptophan hydroxylase gene A218C polymorphism and suicidality in adolescents. *Am J Med Genet* 2001; **105**(5)**:** 451-457.

207. Zalsman G, Frisch A, Lewis R, Michaelovsky E, Hermesh H, Sher L *et al.* DRD4 receptor gene exon III polymorphism in inpatient suicidal adolescents. *J Neural Transm* 2004; **111**(12)**:** 1593-1603.

208. Zalsman G, Patya M, Frisch A, Ofek H, Schapir L, Blum I *et al.* Association of polymorphisms of the serotonergic pathways with clinical traits of impulsive-aggression and suicidality in adolescents: a multi-center study. *World J Biol Psychiatry* 2011; **12**(1)**:** 33-41.

209. Zammit S, Jones G, Jones SJ, Norton N, Sanders RD, Milham C *et al.* Polymorphisms in the MAOA, MAOB, and COMT genes and aggressive behavior in schizophrenia. *Am J Med Genet B Neuropsychiatr Genet* 2004; **128B**(1)**:** 19-20.

210. Zimmermann P, Mohr C, Spangler G. Genetic and attachment influences on adolescents' regulation of autonomy and aggressiveness. *J Child Psychol Psychiatry* 2009; **50**(11)**:** 1339-1347.

211. Zouk H, McGirr A, Lebel V, Benkelfat C, Rouleau G, Turecki G. The effect of genetic variation of the serotonin 1B receptor gene on impulsive aggressive behavior and suicide. *Am J Med Genet B Neuropsychiatr Genet* 2007; **144B**(8)**:** 996-1002.
